# DNA-barcoding of forensically important blow flies (Diptera: Calliphoridae) in the Caribbean Region

**DOI:** 10.7717/peerj.3516

**Published:** 2017-07-25

**Authors:** Sohath Z. Yusseff-Vanegas, Ingi Agnarsson

**Affiliations:** Department of Biology, University of Vermont, Burlington, VT, United States of America

**Keywords:** Calliphoridae, Caribbean, DNA-barcoding, Forensic entomology, Diptera

## Abstract

Correct identification of forensically important insects, such as flies in the family Calliphoridae, is a crucial step for them to be used as evidence in legal investigations. Traditional identification based on morphology has been effective, but has some limitations when it comes to identifying immature stages of certain species. DNA-barcoding, using COI, has demonstrated potential for rapid and accurate identification of Calliphoridae, however, this gene does not reliably distinguish among some recently diverged species, raising questions about its use for delimitation of species of forensic importance. To facilitate DNA based identification of Calliphoridae in the Caribbean we developed a vouchered reference collection from across the region, and a DNA sequence database, and further added the nuclear ITS2 as a second marker to increase accuracy of identification through barcoding. We morphologically identified freshly collected specimens, did phylogenetic analyses and employed several species delimitation methods for a total of 468 individuals representing 19 described species. Our results show that combination of COI + ITS2 genes yields more accurate identification and diagnoses, and better agreement with morphological data, than the mitochondrial barcodes alone. All of our results from independent and concatenated trees and most of the species delimitation methods yield considerably higher diversity estimates than the distance based approach and morphology. Molecular data support at least 24 distinct clades within Calliphoridae in this study, recovering substantial geographic variation for *Lucilia eximia, Lucilia retroversa, Lucilia rica* and *Chloroprocta idioidea*, probably indicating several cryptic species. In sum, our study demonstrates the importance of employing a second nuclear marker for barcoding analyses and species delimitation of calliphorids, and the power of molecular data in combination with a complete reference database to enable identification of taxonomically and geographically diverse insects of forensic importance.

## Introduction

Forensic entomology is the application of the study of insects in legal investigations. Although several groups of insects, mainly of the orders Diptera and Coleoptera, are associated with cadaveric decomposition, blow flies (Diptera: Calliphoridae) are among the most dominant and conspicuous insects in the decomposition process (Catts, 1992). They are useful to determine time of death and, in particular situations, cause of death (Goff, 2000) or relocation of a body (Matuszewski, Szafalowicz & Jarmusz, 2013). During the last five decades of intensive studies in forensic entomology (Byrd & Castner, 2010; Catts & Haskell, 1990; Goff, 2000; Smith, 1986; Tomberlin & Benbow, 2015), the acceptance of insects as evidence in legal investigations has increased gradually and they are now included as standard operating procedures in crime scene investigations in many countries (Tomberlin & Benbow, 2015). Determining the post mortem interval (PMI) is one of the most important tasks during an investigation, and the use of immature stages of Calliphoridae is essential whenever time of death is difficult to establish based on other means (Catts & Haskell, 1990). Although the accurate determination of PMI and period of insect activity (PIA) depend of several factors that are discussed in detail by Catts (1992), the first one, and most important to resolve, is the correct identification of the specimens found at the crime scene. As each species has a specific developmental rate and range of distribution, the accurate identification of insects, mainly the larval stages, is critical because the incorrect determination will invalidate the estimated post mortem interval and impact other interpretations of the evidence (Goff, 2000; Wells & LaMotte, 2001).

Morphology is most commonly used to identify adult insects involved in cadaveric decomposition and taxonomic keys are available for most of the Calliphoridae species. In general, these taxonomic keys include the detailed description of the male and female genitalia, which is examined when external characteristics are not sufficient to establish identity (Tantawi, Whitworth & Sinclair, 2017; Whitworth, 2010; Whitworth, 2014; Whitworth & Rognes, 2012). Identification of immature stages (eggs, larvae and pupae) is more challenging, but possible when detailed taxonomic descriptions exist (Greenberg & Szyska, 1984; Sukontason et al., 2005; Szpila et al., 2013a; Szpila et al., 2014; Szpila & Villet, 2011; Wells, Byrd & Tantawi, 1999). However, in places like the Caribbean, where forensic entomology has not yet been developed, this approach is limited due to the lack of detailed descriptions of immature stages. For instance, from the 18 forensically important calliphorid species currently recognized in the Caribbean, plus the most important livestock pest parasite in the Americas, *C. hominivorax* (Whitworth, 2010), only eight have been documented well enough to be identified based on larvae, mainly using morphology of the third instar (Florez & Wolff, 2009; Wells, Byrd & Tantawi, 1999). For the other species, the identification of immature specimens would need to be done by rearing them to adulthood (Goff, 2000), which is time consuming, may delay legal investigations, and relies on the survival of larvae in the laboratory. Given local endemism, the scarce studies on this group in the Caribbean, and the lack of knowledge of immature stages for at least 11 species, developing alternative tools for identification is important.

With the advances in molecular methods, DNA barcoding has become a widely used technique for species delimitation and identification. This approach allows the identification of specimens during any development stage, including incomplete or damaged specimens, does not require taxonomic expertise, and it is also useful to recognize cryptic species that morphological approaches may not detect (Hebert et al., 2003; Hebert et al., 2004a; Hebert et al., 2004b). Worldwide many authors have used this method to identify species of the family Calliphoridae and these studies showed the potential of the ‘standard barcoding gene’ cytochrome c oxidase subunit I (COI) to distinguish between forensically significant species (Aly & Wen, 2013; Chen et al., 2009; Harvey et al., 2003; Liu et al., 2011; Nelson, Wallman & Dowton, 2007; Wells & Williams, 2007). However, COI does not reliably distinguish among certain closely related calliphorid species, specifically *Chrysomya saffranea* and *Ch. megacephala* (Harvey et al., 2008; Nelson, Wallman & Dowton, 2007), *Ch. semimetalica* and *Ch. latifrons* (Nelson, Wallman & Dowton, 2007), *Calliphora stygia* and *C. albifrontalis, C. dubia* and *C. augur* (Harvey et al., 2008; Wallman & Donnellan, 2001), *C. aldrichia* and *C. montana* (Tantawi, Whitworth & Sinclair, 2017), *Cochliomyia macellaria* and *Co. aldrichi* (Yusseff-Vanegas & Agnarsson, 2016), *Lucilia mexicana* and *L. coeruleiviridis* (DeBry et al., 2013; Whitworth, 2014), *L. bazini* and *L. hainanenesis* (Chen et al., 2014), *L. illustris* and *L. caesar* (Reibe, Schmitz & Madea, 2009; Sonet et al., 2012), *L. cuprina* and *L. sericata* (Williams & Villet, 2013). Given the serious implications of misidentification of forensic insects, an improved protocol for accurate identification is necessary. We propose using the nuclear internal transcribed spacer ITS2 as a second barcoding locus for taxonomic species determinations in calliphorids as suggested by GilArriortua et al. (2014). Although evaluations of ITS2 as unique identification marker have limitations for some taxa (Agnarsson, 2010), several studies have shown the potential application of ITS2 for blowfly species identification (Jordaens et al., 2013a; Nelson, Wallman & Dowton, 2007; Nelson, Wallman & Dowton, 2008, Song, Wang & Liang, 2008; Yusseff-Vanegas & Agnarsson, 2016). We expect a combination of barcodes from the nuclear and mitochondrial genomes to offer a general, simple and reliable way of identifying forensically important insects, even problematic sister species, as successfully done in certain other arthropod groups (Anslan & Tedersoo, 2015; Cao et al., 2016).

The success of DNA barcoding directly links to the quality of the underlying database (Candek & Kuntner, 2015; Coddington et al., 2016; DeBry et al., 2013; Harvey et al., 2003) not only in terms of the quality of identifications but also in terms of taxon sampling (species, geographic localities, populations). Existing efforts in this respect are lacking for Calliphoridae in the Caribbean, limiting the reliability of this technique for delimitation of species. Hitherto, three studies have included molecular data of a few Calliphoridae from the Caribbean (McDonagh, Garcia & Stevens, 2009; Whitworth, 2014; Yusseff-Vanegas & Agnarsson, 2016); they lack the geographic variation necessary to estimate the ratio between intraspecific variation and interspecific divergence from which barcoding accuracy depends (Meyer & Paulay, 2005). Our study provides the first thorough molecular study of Caribbean Calliphoridae.

Our aims are: (1) to establish COI barcode libraries for all Caribbean species and to test if barcodes offer reliable means of their identification, (2) to assess the usefulness of ITS2 as a second barcoding locus in species delimitation and identification, and, (3) to improve online databases with sequences from the Caribbean, including specimens from multiple localities in each island covering the geographic range for each species. To achieve these goals, we sampled 468 specimens of Calliphoridae representing 19 species.

**Table 1:** Specimen details, collection information and GenBank accession numbers. * Estimated coordinate points. ∧ Accession numbers from BOLD systems. – blank.

Genus	Species	Voucher ID	Country	Latitude	Longitude	COI	ITS2
*Calliphora*	*maestrica*	DR084	Hispaniola	N18.82138	W70.67935	MF097182	MF097580
*Calliphora*	*maestrica*	DR085	Hispaniola	N18.82138	W70.67935	MF097183	–
*Calliphora*	*maestrica*	DR086	Hispaniola	N18.82138	W70.67935	MF097184	–
*Calliphora*	*maestrica*	DR087	Hispaniola	N18.82138	W70.67935	MF097185	–
*Calliphora*	*maestrica*	DR088	Hispaniola	N18.82138	W70.67935	MF097186	MF097581
*Chloroprocta*	*idioidea*	CU008	Cuba	N20.054178	W76.917603	MF097187	MF097582
*Chloroprocta*	*idioidea*	CU047	Cuba	N21.582414	W77.783464	MF097188	MF097583
*Chloroprocta*	*idioidea*	CU048	Cuba	N21.582414	W77.783464	MF097189	MF097584
*Chloroprocta*	*idioidea*	CU049	Cuba	N21.582414	W77.783464	MF097190	–
*Chloroprocta*	*idioidea*	DR031	Hispaniola	N18.316572	W71.576447*	MF097191	–
*Chloroprocta*	*idioidea*	DR044	Hispaniola	N18.316572	W71.576447*	MF097192	MF097585
*Chloroprocta*	*idioidea*	DR045	Hispaniola	N18.316572	W71.576447*	MF097193	–
*Chloroprocta*	*idioidea*	DR051	Hispaniola	N19.06753	W69.46445	MF097194	–
*Chloroprocta*	*idioidea*	DR052	Hispaniola	N19.06753	W69.46445	MF097195	MF097586
*Chloroprocta*	*idioidea*	ME001	Mexico	N21.07645	W89.501083	–	MF097587
*Chloroprocta*	*idioidea*	ME002	Mexico	N21.07645	W89.501083	MF097196	MF097588
*Chrysomya*	*albiceps*	CO003	Colombia	N5.900544	W74.852897*	–	MF097589
*Chrysomya*	*albiceps*	CO004	Colombia	N5.900544	W74.852897*	–	MF097590
*Chrysomya*	*albiceps*	CO005	Colombia	N5.900544	W74.852897*	–	MF097591
*Chrysomya*	*albiceps*	LA103	Martinique	N14.47428	W60.81463	MF097199	MF097592
*Chrysomya*	*albiceps*	LA104	Martinique	N14.47428	W60.81463	MF097200	MF097593
*Chrysomya*	*albiceps*	LA125	Saint Lucia	N14.100031	W60.92654	MF097201	MF097594
*Chrysomya*	*albiceps*	LA135	Barbados	N13.2051667	W59.5295556	MF097197	–
*Chrysomya*	*albiceps*	LA136	Barbados	N13.2051667	W59.5295556	MF097198	–
*Chrysomya*	*megacephala*	CO006	Colombia	N5.900544	W74.852897*	MF097202	MF097595
*Chrysomya*	*megacephala*	CO007	Colombia	N5.900544	W74.852897*	–	MF097596
*Chrysomya*	*megacephala*	CO008	Colombia	N6.266242	W77.374903*	MF097203	MF097597
*Chrysomya*	*megacephala*	CO009	Colombia	N5.900544	W74.852897*	–	MF097598
*Chrysomya*	*megacephala*	DR017	Hispaniola	N19.89155	W071.65806	MF097205	–
*Chrysomya*	*megacephala*	DR018	Hispaniola	N19.89155	W071.65806	MF097206	–
*Chrysomya*	*megacephala*	DR068	Hispaniola	N19.06710	W69.46004	MF097207	–
*Chrysomya*	*megacephala*	DR069	Hispaniola	N19.06710	W69.46004	MF097208	–
*Chrysomya*	*megacephala*	DR101	Hispaniola	N18.35698	W68.61609	MF097209	–
*Chrysomya*	*megacephala*	DR102	Hispaniola	N18.35698	W68.61609	MF097210	–
*Chrysomya*	*megacephala*	DR103	Hispaniola	N18.35698	W68.61609	MF097211	–
*Chrysomya*	*megacephala*	DR104	Hispaniola	N18.35698	W68.61609	MF097212	–
*Chrysomya*	*megacephala*	DR116	Hispaniola	N18.32902	W68.80995	MF097213	MF097599
*Chrysomya*	*megacephala*	DR117	Hispaniola	N18.32902	W68.80995	MF097214	MF097611
*Chrysomya*	*megacephala*	DR118	Hispaniola	N18.32902	W68.80995	MF097215	–
*Chrysomya*	*megacephala*	DR119	Hispaniola	N18.32902	W68.80995	MF097216	–
*Chrysomya*	*megacephala*	FL003	Florida, USA	N25.614383	W80.584467	KX529521	KX529561
*Chrysomya*	*megacephala*	FL004	Florida, USA	N25.614383	W80.584467	MF097218	–
*Chrysomya*	*megacephala*	FL011	Florida, USA	N25.086633	W80.452217	MF097219	–
*Chrysomya*	*megacephala*	JA004	Jamaica	N18.0598056	W77.5311944	–	MF097600
*Chrysomya*	*megacephala*	LA062	Dominica	N15.34066	W61.33351	MF097220	–
*Chrysomya*	*megacephala*	LA001	Saint Eustatius	N17.47637	W62.97470	MF097225	–
*Chrysomya*	*megacephala*	LA003	Saint Eustatius	N17.47637	W62.97470	MF097217	–
*Chrysomya*	*megacephala*	LA025	Saint-Martin	N18.07779	W63.05772	MF097235	–
*Chrysomya*	*megacephala*	LA055	Saint Barthélemy	N17.91924	W62.86366	MF097234	–
*Chrysomya*	*megacephala*	LA063	Dominica	N15.34066	W61.33351	MF097204	–
*Chrysomya*	*megacephala*	LA088	Guadeloupe	N16.37752	W61.47869	MF097221	–
*Chrysomya*	*megacephala*	LA089	Guadeloupe	N16.37752	W61.47869	MF097222	–
*Chrysomya*	*megacephala*	LA093	Nevis	N17.14145	W62.57784	MF097226	–
*Chrysomya*	*megacephala*	LA116	Saint Kitts	N17.3404083	W62.7410389	MF097223	–
*Chrysomya*	*megacephala*	LA117	Saint Kitts	N17.3404083	W62.7410389	MF097224	–
*Chrysomya*	*megacephala*	LA123	Saint Lucia	N14.100031	W60.92654	–	MF097604
*Chrysomya*	*megacephala*	ME013	Mexico	N25.598592	W103.441156	–	MF097601
*Chrysomya*	*megacephala*	ME014	Mexico	N25.598592	W103.441156	–	MF097602
*Chrysomya*	*megacephala*	PR038	Puerto Rico	N18.412972	W66.026619	MF097227	–
*Chrysomya*	*megacephala*	PR124	Puerto Rico	N18.370953	W66.026619	MF097228	–
*Chrysomya*	*megacephala*	PR125	Puerto Rico	N18.370953	W66.026619	MF097229	MF097603
*Chrysomya*	*megacephala*	PR1251	Puerto Rico	N18.370953	W66.026619	MF097230	–
*Chrysomya*	*megacephala*	PR126	Puerto Rico	N18.370953	W66.026619	MF097231	–
*Chrysomya*	*megacephala*	PR138	Puerto Rico	N18.447911	W65.948617	MF097232	–
*Chrysomya*	*megacephala*	PR139	Puerto Rico	N18.447911	W65.948617	MF097233	–
*Chrysomya*	*rufifacies*	LA056	Saint Barthélemy	N17.91924	W62.86366	MF097236	–
*Chrysomya*	*rufifacies*	LA057	Saint Barthélemy	N17.91924	W62.86366	MF097237	–
*Chrysomya*	*rufifacies*	CU001	Cuba	N20.054178	W76.917603	MF097238	–
*Chrysomya*	*rufifacies*	CU003	Cuba	N20.054178	W76.917603	MF097239	–
*Chrysomya*	*rufifacies*	CU004	Cuba	N20.054178	W76.917603	KX529555	KX529562
*Chrysomya*	*rufifacies*	CU005	Cuba	N20.054178	W76.917603	MF097240	–
*Chrysomya*	*rufifacies*	CU009	Cuba	N20.054178	W76.917603	MF097241	–
*Chrysomya*	*rufifacies*	CU034	Cuba	N22.621386	W83.725944	MF097242	–
*Chrysomya*	*rufifacies*	CU035	Cuba	N22.621386	W83.725944	MF097243	–
*Chrysomya*	*rufifacies*	CU036	Cuba	N22.621386	W83.725944	MF097244	–
*Chrysomya*	*rufifacies*	CU037	Cuba	N22.621386	W83.725944	MF097245	–
*Chrysomya*	*rufifacies*	DR001	Hispaniola	N19.89155	W71.65806	MF097248	–
*Chrysomya*	*rufifacies*	DR002	Hispaniola	N19.89155	W71.65806	MF097249	–
*Chrysomya*	*rufifacies*	DR003	Hispaniola	N19.89155	W71.65806	MF097250	–
*Chrysomya*	*rufifacies*	DR004	Hispaniola	N19.89155	W71.65806	MF097251	–
*Chrysomya*	*rufifacies*	DR006	Hispaniola	N19.89155	W71.65806	MF097252	–
*Chrysomya*	*rufifacies*	DR007	Hispaniola	N19.89155	W71.65806	MF097253	–
*Chrysomya*	*rufifacies*	DR008	Hispaniola	N19.89155	W71.65806	MF097254	–
*Chrysomya*	*rufifacies*	DR016	Hispaniola	N19.89155	W71.65806	MF097255	–
*Chrysomya*	*rufifacies*	DR036	Hispaniola	N18.316572	W71.576447*	MF097256	–
*Chrysomya*	*rufifacies*	DR037	Hispaniola	N18.316572	W71.576447*	MF097257	–
*Chrysomya*	*rufifacies*	DR038	Hispaniola	N18.316572	W71.576447*	MF097258	–
*Chrysomya*	*rufifacies*	DR039	Hispaniola	N18.316572	W71.576447*	MF097259	–
*Chrysomya*	*rufifacies*	DR070	Hispaniola	N19.06710	W69.46004	MF097260	–
*Chrysomya*	*rufifacies*	DR071	Hispaniola	N19.06710	W69.46004	MF097261	MF097605
*Chrysomya*	*rufifacies*	DR0711	Hispaniola	N19.06710	W69.46004	–	MF097606
*Chrysomya*	*rufifacies*	DR093	Hispaniola	N18.35698	W68.61609	MF097262	–
*Chrysomya*	*rufifacies*	DR094	Hispaniola	N18.35698	W68.61609	MF097263	–
*Chrysomya*	*rufifacies*	DR095	Hispaniola	N18.35698	W68.61609	MF097264	–
*Chrysomya*	*rufifacies*	DR096	Hispaniola	N18.35698	W68.61609	MF097265	–
*Chrysomya*	*rufifacies*	DR097	Hispaniola	N18.35698	W68.61609	MF097266	–
*Chrysomya*	*rufifacies*	DR098	Hispaniola	N18.35698	W68.61609	MF097267	–
*Chrysomya*	*rufifacies*	DR099	Hispaniola	N18.35698	W68.61609	MF097268	–
*Chrysomya*	*rufifacies*	DR100	Hispaniola	N18.35698	W68.61609	MF097269	–
*Chrysomya*	*rufifacies*	DR132	Hispaniola	N18.32902	W68.80995	MF097270	–
*Chrysomya*	*rufifacies*	DR133	Hispaniola	N18.32902	W68.80995	MF097271	–
*Chrysomya*	*rufifacies*	DR135	Hispaniola	N19.741319	W70.654975*	MF097272	–
*Chrysomya*	*rufifacies*	DR150	Hispaniola	N19.34405	W70.14824	MF097273	–
*Chrysomya*	*rufifacies*	DR151	Hispaniola	N19.34405	W70.14824	MF097274	–
*Chrysomya*	*rufifacies*	DR152	Hispaniola	N19.34405	W70.14824	MF097275	–
*Chrysomya*	*rufifacies*	DR155	Hispaniola	N19.34405	W70.14824	MF097276	–
*Chrysomya*	*rufifacies*	DR157	Hispaniola	N18.32902	W68.80995	MF097277	–
*Chrysomya*	*rufifacies*	DR158	Hispaniola	N18.32902	W68.80995	MF097278	–
*Chrysomya*	*rufifacies*	DR159	Hispaniola	N18.32902	W68.80995	MF097279	–
*Chrysomya*	*rufifacies*	DR160	Hispaniola	N18.32902	W68.80995	MF097280	–
*Chrysomya*	*rufifacies*	DR161	Hispaniola	N18.32902	W68.80995	MF097281	–
*Chrysomya*	*rufifacies*	DR162	Hispaniola	N18.32902	W68.80995	MF097282	–
*Chrysomya*	*rufifacies*	DR163	Hispaniola	N18.32902	W68.80995	MF097283	–
*Chrysomya*	*rufifacies*	FL001	Florida, USA	N25.614383	W80.584467	MF097288	–
*Chrysomya*	*rufifacies*	FL010	Florida, USA	N25.086633	W80.452217	MF097289	MF097607
*Chrysomya*	*rufifacies*	JA003	Jamaica	N18.0598056	W77.5311944	MF097293	MF097608
*Chrysomya*	*rufifacies*	LA002	Saint Eustatius	N17.47637	W62.97470	MF097284	–
*Chrysomya*	*rufifacies*	LA004	Saint Eustatius	N17.47637	W62.97470	MF097285	–
*Chrysomya*	*rufifacies*	LA005	Saint Eustatius	N17.47637	W62.97470	MF097286	–
*Chrysomya*	*rufifacies*	LA006	Saint Eustatius	N17.47637	W62.97470	MF097287	–
*Chrysomya*	*rufifacies*	LA041	Saint-Martin	N18.11677	W63.03902	MF097316	–
*Chrysomya*	*rufifacies*	LA042	Saint-Martin	N18.11677	W63.03902	MF097317	–
*Chrysomya*	*rufifacies*	LA043	Saint-Martin	N18.11677	W63.03902	MF097318	–
*Chrysomya*	*rufifacies*	LA044	Saint-Martin	N18.11677	W63.03902	MF097319	–
*Chrysomya*	*rufifacies*	LA069	Dominica	N15.34066	W61.33351	MF097246	–
*Chrysomya*	*rufifacies*	LA072	Dominica	N15.34066	W61.33351	MF097247	–
*Chrysomya*	*rufifacies*	LA090	Guadeloupe	N16.37752	W61.47869	MF097290	–
*Chrysomya*	*rufifacies*	LA091	Guadeloupe	N16.37752	W61.47869	MF097291	–
*Chrysomya*	*rufifacies*	LA092	Guadeloupe	N16.37752	W61.47869	MF097292	–
*Chrysomya*	*rufifacies*	LA101	Martinique	N14.47428	W60.81463	MF097310	–
*Chrysomya*	*rufifacies*	LA108	Montserrat	N16.77608	W62.30904	MF097309	–
*Chrysomya*	*rufifacies*	LA110	Saint Kitts	N17.3404083	W62.7410389	MF097294	MF097609
*Chrysomya*	*rufifacies*	M074	Mona, Puerto Rico	N18.086239	W67.906339	MF097295	–
*Chrysomya*	*rufifacies*	M075	Mona, Puerto Rico	N18.086239	W67.906339	MF097296	–
*Chrysomya*	*rufifacies*	M082	Mona, Puerto Rico	N18.11125	W67.933447	MF097297	–
*Chrysomya*	*rufifacies*	M083	Mona, Puerto Rico	N18.11125	W67.933447	MF097298	–
*Chrysomya*	*rufifacies*	M089	Mona, Puerto Rico	N18.06301	W67.88728	MF097299	–
*Chrysomya*	*rufifacies*	M090	Mona, Puerto Rico	N18.06301	W67.88728	MF097300	–
*Chrysomya*	*rufifacies*	M091	Mona, Puerto Rico	N18.06301	W67.88728	MF097301	–
*Chrysomya*	*rufifacies*	M093	Mona, Puerto Rico	N18.084222	W67.939417	MF097302	–
*Chrysomya*	*rufifacies*	M094	Mona, Puerto Rico	N18.084222	W67.939417	MF097303	–
*Chrysomya*	*rufifacies*	M095	Mona, Puerto Rico	N18.084222	W67.939417	MF097304	–
*Chrysomya*	*rufifacies*	M096	Mona, Puerto Rico	N18.084222	W67.939417	MF097305	–
*Chrysomya*	*rufifacies*	M101	Mona, Puerto Rico	N18.084222	W67.939417	MF097306	–
*Chrysomya*	*rufifacies*	M108	Mona, Puerto Rico	N18.11125	W67.933447	MF097307	–
*Chrysomya*	*rufifacies*	M109	Mona, Puerto Rico	N18.11125	W67.933447	MF097308	–
*Chrysomya*	*rufifacies*	PR117	Puerto Rico	N18.370953	W66.026619	MF097311	–
*Chrysomya*	*rufifacies*	PR118	Puerto Rico	N18.370953	W66.026619	MF097312	–
*Chrysomya*	*rufifacies*	PR119	Puerto Rico	N18.370953	W66.026619	MF097313	–
*Chrysomya*	*rufifacies*	PR120	Puerto Rico	N18.370953	W66.026619	MF097314	–
*Chrysomya*	*rufifacies*	PR130	Puerto Rico	N18.093306	W65.556083	MF097315	MF097610
*Cochliomyia*	*aldrichi*	M080	Mona, Puerto Rico	N18.084222	W65.939417	KX529529	KX529563
*Cochliomyia*	*aldrichi*	M084	Mona, Puerto Rico	N18.11125	W67.933447	MF097320	–
*Cochliomyia*	*aldrichi*	M085	Mona, Puerto Rico	N18.11125	W67.933447	KX529530	KX529564
*Cochliomyia*	*aldrichi*	M086	Mona, Puerto Rico	N18.06301	W67.88728	KX529531	KX529565
*Cochliomyia*	*aldrichi*	M087	Mona, Puerto Rico	N18.06301	W67.88728	MF097321	–
*Cochliomyia*	*aldrichi*	M088	Mona, Puerto Rico	N18.06301	W67.88728	MF097322	–
*Cochliomyia*	*aldrichi*	M102	Mona, Puerto Rico	N18.11125	W67.933447	MF097323	–
*Cochliomyia*	*aldrichi*	M103	Mona, Puerto Rico	N18.11125	W67.933447	KX529532	KX529566
*Cochliomyia*	*aldrichi*	M104	Mona, Puerto Rico	N18.085972	W67.933447	MF097324	–
*Cochliomyia*	*aldrichi*	M105	Mona, Puerto Rico	N18.085972	W67.933447	KX529533	KX529567
*Cochliomyia*	*aldrichi*	M106	Mona, Puerto Rico	N18.084222	W67.939417	MF097325	–
*Cochliomyia*	*aldrichi*	M107	Mona, Puerto Rico	N18.084222	W67.939417	KX529534	KX529568
*Cochliomyia*	*hominivorax*	CO001	Colombia	N5.900544	W74.852897*	–	MF097612
*Cochliomyia*	*hominivorax*	CU020	Cuba	N22.621386	W83.725944	–	MF097613
*Cochliomyia*	*hominivorax*	CU033	Cuba	N22.621386	W83.725944	KX529556	KX529571
*Cochliomyia*	*hominivorax*	DR042	Hispaniola	N18.316572	W71.576447*	KX529557	KX529572
*Cochliomyia*	*hominivorax*	DR105	Hispaniola	N18.35698	W68.61609	KX529558	KX529573
*Cochliomyia*	*macellaria*	LA137	Saint Barthélemy	N17.910299	W62.847221	MF097326	–
*Cochliomyia*	*macellaria*	LA139	Saint Barthélemy	N17.910299	W62.847221	MF097327	–
*Cochliomyia*	*macellaria*	CO002	Colombia	N5.900544	W74.852897*	KX529522	KX529574
*Cochliomyia*	*macellaria*	CO010	Colombia	N6.266242	W77.374903*	KX529545	KX529575
*Cochliomyia*	*macellaria*	CU012	Cuba	N22.621386	W83.725944	MF097330	–
*Cochliomyia*	*macellaria*	CU013	Cuba	N22.621386	W83.725944	MF097331	–
*Cochliomyia*	*macellaria*	CU014	Cuba	N22.621386	W83.725944	KX529541	KX529577
*Cochliomyia*	*macellaria*	CU015	Cuba	N22.621386	W83.725944	MF097332	–
*Cochliomyia*	*macellaria*	CU016	Cuba	N22.621386	W83.725944	MF097333	–
*Cochliomyia*	*macellaria*	CU017	Cuba	N22.621386	W83.725944	MF097334	–
*Cochliomyia*	*macellaria*	CU018	Cuba	N22.621386	W83.725944	KX529526	KX529578
*Cochliomyia*	*macellaria*	CU019	Cuba	N22.621386	W83.725944	MF097335	MF097614
*Cochliomyia*	*macellaria*	CU050	Cuba	N21.582414	W77.750131	MF097336	–
*Cochliomyia*	*macellaria*	CU051	Cuba	N21.582414	W77.750131	MF097337	–
*Cochliomyia*	*macellaria*	DR009	Hispaniola	N19.89155	W71.65806	MF097341	–
*Cochliomyia*	*macellaria*	DR010	Hispaniola	N19.89155	W71.65806	KX529536	KX529579
*Cochliomyia*	*macellaria*	DR011	Hispaniola	N19.89155	W71.65806	MF097342	–
*Cochliomyia*	*macellaria*	DR012	Hispaniola	N19.89155	W71.65806	MF097343	–
*Cochliomyia*	*macellaria*	DR013	Hispaniola	N19.89155	W71.65806	MF097344	–
*Cochliomyia*	*macellaria*	DR014	Hispaniola	N19.89155	W71.65806	MF097345	–
*Cochliomyia*	*macellaria*	DR015	Hispaniola	N19.89155	W71.65806	MF097346	–
*Cochliomyia*	*macellaria*	DR043	Hispaniola	N18.316572	W71.576447*	MF097347	–
*Cochliomyia*	*macellaria*	DR062	Hispaniola	N19.06710	W69.46004	MF097348	–
*Cochliomyia*	*macellaria*	DR063	Hispaniola	N19.06710	W69.46004	MF097349	–
*Cochliomyia*	*macellaria*	DR064	Hispaniola	N19.06710	W69.46004	MF097350	–
*Cochliomyia*	*macellaria*	DR065	Hispaniola	N19.06710	W69.46004	MF097351	–
*Cochliomyia*	*macellaria*	DR066	Hispaniola	N19.06710	W69.46004	MF097352	–
*Cochliomyia*	*macellaria*	DR106	Hispaniola	N18.35698	W68.61609	MF097353	–
*Cochliomyia*	*macellaria*	DR107	Hispaniola	N18.35698	W68.61609	MF097354	–
*Cochliomyia*	*macellaria*	DR108	Hispaniola	N18.35698	W68.61609	MF097355	–
*Cochliomyia*	*macellaria*	DR109	Hispaniola	N18.35698	W68.61609	MF097356	–
*Cochliomyia*	*macellaria*	DR1091	Hispaniola	N18.35698	W68.61609	MF097357	–
*Cochliomyia*	*macellaria*	DR120	Hispaniola	N18.32902	W68.80995	MF097358	–
*Cochliomyia*	*macellaria*	DR121	Hispaniola	N18.32902	W68.80995	MF097359	–
*Cochliomyia*	*macellaria*	DR134	Hispaniola	N19.741319	W70.654975*	KX529527	KX529580
*Cochliomyia*	*macellaria*	DR154	Hispaniola	N19.34405	W70.14824	MF097360	–
*Cochliomyia*	*macellaria*	FL006	Florida, USA	N25.614383	W80.584467	–	MF097615
*Cochliomyia*	*macellaria*	FL009	Florida, USA	N25.457514	W80.4863	MF097361	–
*Cochliomyia*	*macellaria*	JA002	Jamaica	N18.0598056	W77.5311944	–	MF097616
*Cochliomyia*	*macellaria*	LA022	Saint-Martin	N18.07779	W63.05772	MF097384	–
*Cochliomyia*	*macellaria*	LA023	Saint-Martin	N18.07779	W63.05772	MF097385	–
*Cochliomyia*	*macellaria*	LA024	Saint-Martin	N18.07779	W63.05772	MF097386	–
*Cochliomyia*	*macellaria*	LA032	Saint-Martin	N18.11677	W63.03902	MF097387	–
*Cochliomyia*	*macellaria*	LA033	Saint-Martin	N18.11677	W63.03902	MF097388	–
*Cochliomyia*	*macellaria*	LA034	Saint-Martin	N18.11677	W63.03902	MF097389	–
*Cochliomyia*	*macellaria*	LA035	Saint-Martin	N18.11677	W63.03902	MF097390	–
*Cochliomyia*	*macellaria*	LA036	Saint-Martin	N18.11677	W63.03902	MF097391	–
*Cochliomyia*	*macellaria*	LA049	Saint Barthélemy	N17.91924	W62.86366	MF097371	–
*Cochliomyia*	*macellaria*	LA0491	Saint Barthélemy	N17.91924	W62.86366	MF097372	–
*Cochliomyia*	*macellaria*	LA050	Saint Barthélemy	N17.91924	W62.86366	MF097373	–
*Cochliomyia*	*macellaria*	LA053	Saint Barthélemy	N17.91924	W62.86366	MF097383	–
*Cochliomyia*	*macellaria*	LA054	Saint Barthélemy	N17.91924	W62.86366	MF097374	–
*Cochliomyia*	*macellaria*	LA066	Dominica	N15.34066	W61.33351	MF097338	–
*Cochliomyia*	*macellaria*	LA067	Dominica	N15.34066	W61.33351	MF097339	–
*Cochliomyia*	*macellaria*	LA068	Dominica	N15.34066	W61.33351	MF097340	–
*Cochliomyia*	*macellaria*	LA071	Dominica	N15.34066	W61.33351	KX529525	KX529583
*Cochliomyia*	*macellaria*	LA079	Guadeloupe	N16.37752	W61.47869	MF097362	–
*Cochliomyia*	*macellaria*	LA080	Guadeloupe	N16.37752	W61.47869	MF097363	–
*Cochliomyia*	*macellaria*	LA081	Guadeloupe	N16.37752	W61.47869	MF097364	–
*Cochliomyia*	*macellaria*	LA094	Nevis	N17.14145	W62.57784	MF097368	–
*Cochliomyia*	*macellaria*	LA096	Martinique	N14.47428	W60.81463	KX529524	KX529584
*Cochliomyia*	*macellaria*	LA097	Martinique	N14.47428	W60.81463	MF097367	–
*Cochliomyia*	*macellaria*	LA115	Saint Kitts	N17.3404083	W62.7410389	MF097365	–
*Cochliomyia*	*macellaria*	LA118	Saint Kitts	N17.3404083	W62.7410389	MF097392	–
*Cochliomyia*	*macellaria*	LA131	Barbuda	N17.6054722	W61.8005833	MF097328	–
*Cochliomyia*	*macellaria*	LA132	Barbuda	N17.6054722	W61.8005833	MF097329	–
*Cochliomyia*	*macellaria*	LA138	Saint Barthélemy	N17.897522	W62.849694	MF097375	–
*Cochliomyia*	*macellaria*	LA140	Saint Barthélemy	N17.897522	W62.849694	MF097376	–
*Cochliomyia*	*macellaria*	LA141	Saint Barthélemy	N17.897522	W62.849694	MF097377	–
*Cochliomyia*	*macellaria*	LA142	Saint Barthélemy	N17.897522	W62.849694	KX529523	KX529592
*Cochliomyia*	*macellaria*	LA143	Saint Barthélemy	N17.897522	W62.849694	MF097378	–
*Cochliomyia*	*macellaria*	LA144	Saint Barthélemy	N17.897522	W62.849694	MF097379	–
*Cochliomyia*	*macellaria*	LA145	Saint Barthélemy	N17.897522	W62.849694	MF097380	–
*Cochliomyia*	*macellaria*	LA146	Saint Barthélemy	N17.897522	W62.849694	MF097381	–
*Cochliomyia*	*macellaria*	LA147	Saint Barthélemy	N17.897522	W62.849694	MF097382	
*Cochliomyia*	*macellaria*	ME015	Mexico	N25.598592	W103.441156	–	MF097617
*Cochliomyia*	*macellaria*	M077	Mona, Puerto Rico	N18.086239	W67.906339	KX529539	KX529585
*Cochliomyia*	*macellaria*	M081	Mona, Puerto Rico	N18.11125	W67.933447	KX529537	KX529586
*Cochliomyia*	*macellaria*	M112	Mona, Puerto Rico	N18.11125	W67.933447	KX529544	KX529589
*Cochliomyia*	*macellaria*	ME004	Mexico	N21.07645	W89.501083	MF097366	–
*Cochliomyia*	*macellaria*	PR029	Puerto Rico	N17.961111	W66.863806	MF097369	–
*Cochliomyia*	*macellaria*	PR047	Puerto Rico	N18.178722	W66.488111	MF097370	–
*Cochliomyia*	*macellaria*	PR121	Puerto Rico	N18.370953	W66.026619	KX529544	KX529589
*Cochliomyia*	*macellaria*	PR128	Puerto Rico	N18.093306	W65.552111	KX529540	KX529590
*Cochliomyia*	*macellaria*	PR129	Puerto Rico	N18.093306	W65.552111	KX529542	KX529591
*Cochliomyia*	*minima*	CU010	Cuba	N20.054178	W76.917603	MF097393	–
*Cochliomyia*	*minima*	CU021	Cuba	N22.621386	W83.725944	MF097394	–
*Cochliomyia*	*minima*	CU022	Cuba	N22.621386	W83.725944	KX529549	KX529593
*Cochliomyia*	*minima*	CU023	Cuba	N22.621386	W83.725944	KX529550	KX529594
*Cochliomyia*	*minima*	CU024	Cuba	N22.621386	W83.725944	MF097395	–
*Cochliomyia*	*minima*	CU025	Cuba	N22.621386	W83.725944	MF097396	–
*Cochliomyia*	*minima*	CU026	Cuba	N22.621386	W83.725944	MF097397	–
*Cochliomyia*	*minima*	CU027	Cuba	N22.621386	W83.725944	MF097398	–
*Cochliomyia*	*minima*	CU043	Cuba	N20.517817	W74.65865	MF097399	–
*Cochliomyia*	*minima*	CU044	Cuba	N20.517817	W74.65865	MF097400	–
*Cochliomyia*	*minima*	CU045	Cuba	N20.517817	W74.65865	MF097401	–
*Cochliomyia*	*minima*	CU046	Cuba	N20.517817	W74.65865	KX529547	KX529595
*Cochliomyia*	*minima*	DR026	Hispaniola	N19.04995	W70.89046	MF097402	–
*Cochliomyia*	*minima*	DR027	Hispaniola	N19.04995	W70.89046	MF097403	–
*Cochliomyia*	*minima*	DR028	Hispaniola	N19.04995	W70.89046	MF097404	–
*Cochliomyia*	*minima*	DR029	Hispaniola	N19.04995	W70.89046	MF097405	–
*Cochliomyia*	*minima*	PR013	Hispaniola	N18.316572	W71.576447*	MF097406	–
*Cochliomyia*	*minima*	DR032	Hispaniola	N18.316572	W71.576447*	MF097407	–
*Cochliomyia*	*minima*	DR033	Hispaniola	N18.316572	W71.576447*	MF097408	–
*Cochliomyia*	*minima*	DR034	Hispaniola	N18.316572	W71.576447*	MF097409	–
*Cochliomyia*	*minima*	DR035	Hispaniola	N18.316572	W71.576447*	MF097410	–
*Cochliomyia*	*minima*	DR053	Hispaniola	N19.06753	W69.46445	MF097411	–
*Cochliomyia*	*minima*	DR054	Hispaniola	N19.06753	W69.46445	MF097412	–
*Cochliomyia*	*minima*	DR055	Hispaniola	N19.06753	W69.46445	KX529552	KX529596
*Cochliomyia*	*minima*	DR056	Hispaniola	N19.06753	W69.46445	MF097413	–
*Cochliomyia*	*minima*	DR067	Hispaniola	N19.06710	W69.46004	MF097414	–
*Cochliomyia*	*minima*	DR072	Hispaniola	N19.34864	W70.14910	MF097415	–
*Cochliomyia*	*minima*	DR073	Hispaniola	N19.34864	W70.14910	MF097416	–
*Cochliomyia*	*minima*	DR074	Hispaniola	N19.34864	W70.14910	MF097417	–
*Cochliomyia*	*minima*	DR075	Hispaniola	N19.34864	W70.14910	MF097418	–
*Cochliomyia*	*minima*	DR076	Hispaniola	N19.34864	W70.14910	MF097419	–
*Cochliomyia*	*minima*	DR136	Hispaniola	N19.741319	W70.654975	KX529548	KX529597
*Cochliomyia*	*minima*	DR137	Hispaniola	N19.741319	W70.654975	MF097420	–
*Cochliomyia*	*minima*	DR138	Hispaniola	N19.741319	W70.654975	MF097421	–
*Cochliomyia*	*minima*	DR139	Hispaniola	N19.741319	W70.654975	MF097422	–
*Cochliomyia*	*minima*	DR153	Hispaniola	N19.34405	W70.14824	MF097423	–
*Cochliomyia*	*minima*	DR164	Hispaniola	N18.32902	W68.80995	MF097424	–
*Cochliomyia*	*minima*	PR006	Puerto Rico	N18.412972	W66.727222	MF097425	–
*Cochliomyia*	*minima*	PR007	Puerto Rico	N18.412972	W66.727222	MF097426	–
*Cochliomyia*	*minima*	PR016	Puerto Rico	N18.321333	W65.818722	MF097427	–
*Cochliomyia*	*minima*	PR018	Puerto Rico	N18.321333	W65.818722	MF097428	–
*Cochliomyia*	*minima*	PR019	Puerto Rico	N18.321333	W65.818722	MF097429	–
*Cochliomyia*	*minima*	PR041	Puerto Rico	N18.174722	W66.491861	MF097430	–
*Cochliomyia*	*minima*	PR131	Puerto Rico	N18.093306	W65.552111	MF097431	–
*Cochliomyia*	*minima*	PR132	Puerto Rico	N18.093306	W65.552111	KX529553	KX529598
*Cochliomyia*	*minima*	PR133	Puerto Rico	N18.093306	W65.552111	KX529554	KX529599
*Cochliomyia*	*minima*	PR140	Puerto Rico	N18.447911	W65.948617	MF097432	MF097618
*Cochliomyia*	*minima*	PR141	Puerto Rico	N18.447911	W65.948617	KX529551	KX529600
*Cochliomyia*	*minima*	PR145	Puerto Rico	N18.449889	W65.595333	MF097433	–
*Cochliomyia*	*minima*	PR146	Puerto Rico	N18.449889	W65.595333	MF097434	–
*Lucilia*	*cluvia*	FL005	Florida, USA	N25.614383	W80.584467	–	MF097619
*Lucilia*	*cluvia*	FL017	Florida, USA	N25.136917	W80.94855	MF097436	MF097620
*Lucilia*	*cluvia*	FL018	Florida, USA	N25.136917	W80.94855	–	MF097621
*Lucilia*	*cluvia*	FL019	Florida, USA	N25.323331	W80.833094	MF097437	–
*Lucilia*	*cluvia*	FL020	Florida, USA	N25.323331	W80.833094	MF097438	MF097622
*Lucilia*	*cluvia*	FL025	Florida, USA	N25.423053	W80.679114	MF097439	MF097623
*Lucilia*	*cluvia*	FL026	Florida, USA	N25.423053	W80.679114	MF097440	MF097624
*Lucilia*	*cluvia*	PR147	Puerto Rico	N18.429222	W66.178022	MF097441	MF097625
*Lucilia*	*cluvia*	PR148	Puerto Rico	N18.429222	W66.178022	MF097442	MF097626
*Lucilia*	*coeruleiviridis*	FL007	Florida, USA	N25.457514	W80.4863	–	MF097627
*Lucilia*	*coeruleiviridis*	FL013	Florida, USA	N25.136917	W80.94885	MF097443	MF097628
*Lucilia*	*coeruleiviridis*	FL014	Florida, USA	N25.136917	W80.94855	–	MF097629
*Lucilia*	*coeruleiviridis*	FL015	Florida, USA	N25.136917	W80.94885	MF097444	MF097630
*Lucilia*	*coeruleiviridis*	FL016	Florida, USA	N25.136917	W80.94885	MF097445	MF097631
*Lucilia*	*coeruleiviridis*	FL023	Florida, USA	N25.457514	W80.4863	MF097446	MF097632
*Lucilia*	*coeruleiviridis*	FL024	Florida, USA	N25.457514	W80.4863	MF097447	MF097633
*Lucilia*	*cuprina*	FL027	Florida, USA	N25.457514	W80.4863	MF097448	MF097634
*Lucilia*	*cuprina*	FL028	Florida, USA	N25.457514	W80.4863	MF097449	MF097635
*Lucilia*	*cuprina*	FL029	Florida, USA	N25.457514	W80.4863	MF097450	MF097636
*Lucilia*	*cuprina*	FL030	Florida, USA	N25.457514	W80.4863	MF097451	MF097637
*Lucilia*	*cuprina*	PR070	Puerto Rico	N18.370953	W66.026619	MF097452	–
*Lucilia*	*cuprina*	PR071	Puerto Rico	N18.370953	W66.026619	MF097453	–
*Lucilia*	*cuprina*	PR072	Puerto Rico	N18.370953	W66.026619	MF097454	–
*Lucilia*	*cuprina*	PR073	Puerto Rico	N18.370953	W66.026619	KX529559	KX529602
*Lucilia*	*cuprina*	PR122	Puerto Rico	N18.370953	W66.026619	MF097455	MF097638
*Lucilia*	*cuprina*	PR123	Puerto Rico	N18.370953	W66.026619	MF097456	–
*Lucilia*	*cuprina*	PR153	Puerto Rico	N18.461053	W66.729803	MF097457	–
*Lucilia*	*cuprina*	PR154	Puerto Rico	N18.461053	W66.729803	MF097458	MF097639
*Lucilia*	*eximia*	CO011	Colombia	N5.900544	W74.852897*	MF097459	–
*Lucilia*	*eximia*	CO012	Colombia	N5.900544	W74.852897*	MF097460	MF097640
*Lucilia*	*eximia*	CO013	Colombia	N5.900544	W74.852897*	MF097461	MF097641
*Lucilia*	*eximia*	CO015	Colombia	N5.900544	W74.852897*	MF097462	MF097642
*Lucilia*	*eximia*	CO016	Colombia	N5.900544	W74.852897*	–	MF097643
*Lucilia*	*eximia*	CO022	Colombia	N6.067217	W73.645411	MF097463	MF097644
*Lucilia*	*eximia*	CO023	Colombia	N6.067217	W73.645411	MF097464	MF097645
*Lucilia*	*eximia*	CU002	Cuba	N20.054178	W76.917603	–	MF097646
*Lucilia*	*eximia*	CU006	Cuba	N20.054178	W76.917603	–	MF097647
*Lucilia*	*eximia*	DR019	Hispaniola	N19.89155	W071.65806	MF097467	MF097650
*Lucilia*	*eximia*	DR049	Hispaniola	N18.316572	W71.576447*	MF097468	–
*Lucilia*	*eximia*	DR050	Hispaniola	N18.316572	W71.576447	–	MF097651
*Lucilia*	*eximia*	DR129	Hispaniola	N18.32902	W68.80995	MF097469	–
*Lucilia*	*eximia*	FL021	Florida, USA	N25.086633	W80.452217	MF097470	MF097652
*Lucilia*	*eximia*	FL022	Florida, USA	N25.086633	W80.452217	MF097471	MF097653
*Lucilia*	*eximia*	LA064	Dominica	N15.34066	W61.33351	MF097465	MF097648
*Lucilia*	*eximia*	LA065	Dominica	N15.34066	W61.33351	MF097466	MF097649
*Lucilia*	*eximia*	LA124	Saint Lucia	N14.100031	W60.92654	MF097483	MF097665
*Lucilia*	*eximia*	LA126	Saint Lucia	N14.100031	W60.92654	–	MF097666
*Lucilia*	*eximia*	LA127	Saint Lucia	N14.100031	W60.92654	MF097484	MF097667
*Lucilia*	*eximia*	M076	Mona, Puerto Rico	N18.086239	W67.906339	MF097472	MF097654
*Lucilia*	*eximia*	M099	Mona, Puerto Rico	N18.084222	W67.939417	MF097473	–
*Lucilia*	*eximia*	M100	Mona, Puerto Rico	N18.084222	W67.939417	MF097474	–
*Lucilia*	*eximia*	M110	Mona, Puerto Rico	N18.11125	W67.933447	MF097475	MF097655
*Lucilia*	*eximia*	M111	Mona, Puerto Rico	N18.11125	W67.933447	MF097476	MF097656
*Lucilia*	*eximia*	ME005	Mexico	N21.07645	W89.501083	MF097477	MF097657
*Lucilia*	*eximia*	ME006	Mexico	N21.07645	W89.501083	–	MF097658
*Lucilia*	*eximia*	ME007	Mexico	N21.07645	W89.501083	MF097478	MF097659
*Lucilia*	*eximia*	PR050	Puerto Rico	N18.449889	W66.595333	MF097479	MF097660
*Lucilia*	*eximia*	PR060	Puerto Rico	N17.971611	W66.865361	MF097480	MF097661
*Lucilia*	*eximia*	PR111	Mona, Puerto Rico	N18.11125	W67.933447	–	MF097662
*Lucilia*	*eximia*	PR114	Puerto Rico	N18.370953	W66.026619	MF097481	–
*Lucilia*	*eximia*	PR134	Puerto Rico	N18.093306	W65.552111	–	MF097663
*Lucilia*	*eximia*	PR135	Puerto Rico	N18.093306	W65.552111	–	MF097664
*Lucilia*	*eximia*	PR150	Puerto Rico	N18.084222	W67.939417	MF097482	–
*Lucilia*	*fayeae*	M079	Mona, Puerto Rico	N18.084222	W67.939417	MF097485	MF097668
*Lucilia*	*fayeae*	PR008	Puerto Rico	N18.412972	W67.727222	MF097486	MF097669
*Lucilia*	*fayeae*	PR012	Puerto Rico	N18.412972	W67.727222	MF097487	–
*Lucilia*	*fayeae*	PR020	Puerto Rico	N18.321333	W65.818722	MF097488	–
*Lucilia*	*fayeae*	PR022	Puerto Rico	N18.321333	W65.818722	MF097489	–
*Lucilia*	*fayeae*	PR023	Puerto Rico	N18.293444	W65.791917	MF097490	MF097670
*Lucilia*	*fayeae*	PR045	Puerto Rico	N18.174722	W66.491861	MF097491	MF097671
*Lucilia*	*fayeae*	PR053	Puerto Rico	N18.449889	W66.595333	MF097492	MF097672
*Lucilia*	*fayeae*	PR116	Puerto Rico	N18.370953	W66.032175	MF097493	–
*Lucilia*	*lucigerens*	JA005	Jamaica	N18.0598056	W77.5311944	MF097494	MF097673
*Lucilia*	*lucigerens*	JA006	Jamaica	N18.0598056	W77.5311944	MF097495	–
*Lucilia*	*lucigerens*	JA007	Jamaica	N18.0598056	W77.5311944	MF097496	MF097674
*Lucilia*	*mexicana*	ME016	Mexico	N25.598592	W103.441156	MF097497	MF097675
*Lucilia*	*mexicana*	ME020	Mexico	N25.598592	W103.441156	MF097498	MF097676
*Lucilia*	*mexicana*	ME021	Mexico	N25.598592	W103.441156	MF097499	MF097677
*Lucilia*	*retroversa*	CU007	Cuba	N20.054178	W76.917603	MF097500	MF097678
*Lucilia*	*retroversa*	CU028	Cuba	N22.621386	W83.725944	MF097501	–
*Lucilia*	*retroversa*	CU029	Cuba	N22.621386	W83.725944	MF097502	–
*Lucilia*	*retroversa*	CU030	Cuba	N22.621386	W83.725944	MF097503	MF097679
*Lucilia*	*retroversa*	CU031	Cuba	N22.621386	W83.725944	MF097504	–
*Lucilia*	*retroversa*	CU038	Cuba	N20.517817	W20.517817	MF097505	–
*Lucilia*	*retroversa*	CU039	Cuba	N20.517817	W20.517817	MF097506	–
*Lucilia*	*retroversa*	CU040	Cuba	N20.517817	W20.517817	MF097507	–
*Lucilia*	*retroversa*	CU041	Cuba	N20.517817	W20.517817	MF097508	MF097680
*Lucilia*	*retroversa*	CU042	Cuba	N20.517817	W20.517817	MF097509	–
*Lucilia*	*retroversa*	DR020	Hispaniola	N19.04871	W70.88084	MF097510	–
*Lucilia*	*retroversa*	DR021	Hispaniola	N19.04871	W70.88084	MF097511	–
*Lucilia*	*retroversa*	DR022	Hispaniola	N19.04871	W70.88084	MF097512	–
*Lucilia*	*retroversa*	DR023	Hispaniola	N19.04871	W70.88084	MF097513	–
*Lucilia*	*retroversa*	DR024	Hispaniola	N19.04871	W70.88084	MF097514	MF097681
*Lucilia*	*retroversa*	DR025	Hispaniola	N19.04871	W70.88084	MF097515	–
*Lucilia*	*retroversa*	DR030	Hispaniola	N19.04871	W70.88084	MF097516	–
*Lucilia*	*retroversa*	DR040	Hispaniola	N18.316572	W71.576447	MF097517	–
*Lucilia*	*retroversa*	DR046	Hispaniola	N18.316572	W71.576447	MF097518	–
*Lucilia*	*retroversa*	DR047	Hispaniola	N18.316572	W71.576447	MF097519	–
*Lucilia*	*retroversa*	DR048	Hispaniola	N18.316572	W71.576447	MF097520	–
*Lucilia*	*retroversa*	DR057	Hispaniola	N19.06753	W69.46445	MF097521	–
*Lucilia*	*retroversa*	DR058	Hispaniola	N19.06753	W69.46445	MF097522	–
*Lucilia*	*retroversa*	DR059	Hispaniola	N19.06753	W69.46445	MF097523	–
*Lucilia*	*retroversa*	DR060	Hispaniola	N19.06753	W69.46445	MF097524	–
*Lucilia*	*retroversa*	DR061	Hispaniola	N19.06753	W69.46445	MF097525	–
*Lucilia*	*retroversa*	DR079	Hispaniola	N19.34864	W70.14910	MF097526	–
*Lucilia*	*retroversa*	DR080	Hispaniola	N19.34864	W70.14910	MF097527	–
*Lucilia*	*retroversa*	DR081	Hispaniola	N19.34864	W70.14910	MF097528	–
*Lucilia*	*retroversa*	DR082	Hispaniola	N19.34864	W70.14910	MF097529	–
*Lucilia*	*retroversa*	DR083	Hispaniola	N19.34864	W70.14910	MF097530	–
*Lucilia*	*retroversa*	DR089	Hispaniola	N19.34864	W70.14910	MF097531	–
*Lucilia*	*retroversa*	DR090	Hispaniola	N19.34864	W70.14910	MF097532	–
*Lucilia*	*retroversa*	DR091	Hispaniola	N19.34864	W70.14910	MF097533	–
*Lucilia*	*retroversa*	DR092	Hispaniola	N19.34864	W70.14910	MF097534	–
*Lucilia*	*retroversa*	DR111	Hispaniola	N18.35698	W68.61609	MF097535	–
*Lucilia*	*retroversa*	DR110	Hispaniola	N18.35698	W68.61609	MF097536	–
*Lucilia*	*retroversa*	DR112	Hispaniola	N18.35698	W68.61609	MF097537	–
*Lucilia*	*retroversa*	DR122	Hispaniola	N18.32902	W68.80995	MF097538	–
*Lucilia*	*retroversa*	DR123	Hispaniola	N18.32902	W68.80995	MF097539	MF097682
*Lucilia*	*retroversa*	DR124	Hispaniola	N18.32902	W68.80995	MF097540	MF097683
*Lucilia*	*retroversa*	DR125	Hispaniola	N18.32902	W68.80995	MF097541	–
*Lucilia*	*retroversa*	DR126	Hispaniola	N18.32902	W68.80995	MF097542	–
*Lucilia*	*retroversa*	DR128	Hispaniola	N18.32902	W68.80995	MF097543	–
*Lucilia*	*retroversa*	DR140	Hispaniola	N19.741319	W70.654975*	MF097544	–
*Lucilia*	*retroversa*	DR141	Hispaniola	N19.741319	W70.654975*	MF097545	–
*Lucilia*	*retroversa*	DR142	Hispaniola	N18.09786	W71.18925	MF097546	–
*Lucilia*	*retroversa*	DR143	Hispaniola	N18.09786	W71.18925	MF097547	–
*Lucilia*	*retroversa*	DR144	Hispaniola	N18.09786	W71.18925	MF097548	
*Lucilia*	*retroversa*	DR145	Hispaniola	N18.09786	W71.18925	MF097549	–
*Lucilia*	*retroversa*	DR146	Hispaniola	N18.09786	W71.18925	MF097550	–
*Lucilia*	*retroversa*	DR147	Hispaniola	N18.09786	W71.18925	MF097551	–
*Lucilia*	*retroversa*	DR148	Hispaniola	N18.09786	W71.18925	MF097552	–
*Lucilia*	*rica*	LA007	Saint Eustatius	N17.47637	W62.97470	MF097558	–
*Lucilia*	*rica*	LA008	Saint Eustatius	N17.47637	W62.97470	MF097559	–
*Lucilia*	*rica*	LA009	Saint Eustatius	N17.47637	W62.97470	–	MF097684
*Lucilia*	*rica*	LA010	Saint Eustatius	N17.47637	W62.97470	MF097560	–
*Lucilia*	*rica*	LA016	Saint-Martin	N18.07779	W63.05772	MF097572	–
*Lucilia*	*rica*	LA017	Saint-Martin	N18.07779	W63.05772	MF097573	MF097697
*Lucilia*	*rica*	LA026	Saba	N17.63980	W63.23373	MF097435	–
*Lucilia*	*rica*	LA027	Saba	N17.63980	W63.23373	–	MF097692
*Lucilia*	*rica*	LA028	Saba	N18.07779	W63.05772	MF097569	MF097693
*Lucilia*	*rica*	LA037	Saint-Martin	N18.11677	W63.03902	MF097574	–
*Lucilia*	*rica*	LA045	Saint Barthélemy	N17.91924	W62.86366	MF097570	MF097694
*Lucilia*	*rica*	LA061	Saint Barthélemy	N17.91924	W62.86366	MF097571	MF097696
*Lucilia*	*rica*	LA073	Nevis	N17.14145	W62.57784	MF097567	MF097690
*Lucilia*	*rica*	LA074	Nevis	N17.14145	W62.57784	MF097568	MF097691
*Lucilia*	*rica*	LA098	Martinique	N14.47428	W60.81463	MF097565	MF097688
*Lucilia*	*rica*	LA099	Martinique	N14.47428	W60.81463	MF097566	MF097689
*Lucilia*	*rica*	LA106	Montserrat	N16.77608	W62.30904	MF097564	MF097687
*Lucilia*	*rica*	LA114	Saint Kitts	N17.3404083	W62.7410389	MF097563	–
*Lucilia*	*rica*	LA128	Antigua	N17.0358611	W61.8246389	MF097553	–
*Lucilia*	*rica*	LA129	Antigua	N17.0358611	W61.8246389	MF097554	–
*Lucilia*	*rica*	LA130	Antigua	N17.0358611	W61.8246389	MF097555	–
*Lucilia*	*rica*	LA133	Barbuda	N17.6054722	W61.8005833	MF097556	–
*Lucilia*	*rica*	LA134	Barbuda	N17.6054722	W61.8005833	MF097557	–
*Lucilia*	*rica*	LA083	Guadeloupe	N16.37752	W61.47869	MF097561	MF097685
*Lucilia*	*rica*	LA087	Guadeloupe	N16.37752	W61.47869	MF097562	MF097686
*Lucilia*	*rica*	TLW042	Antigua and Barbuda	As published^a^		BNNR042∧	–
*Lucilia*	*rica*	TLW043	Antigua and Barbuda	As published^a^		BNNR043∧	–
*Lucilia*	*rica*	TLW044	Antigua and Barbuda	As published^a^		BNNR044∧	–
*Lucilia*	*rica*	TLW046	Antigua and Barbuda	As published^a^		BNNR046∧	–
*Lucilia*	*sp.*	CO027	Colombia	N6.067217	W73.645411	MF097575	MF097698
*Lucilia*	*vulgata*	CO019	Colombia	N6.067217	W73.645411	MF097576	MF097699
*Lucilia*	*vulgata*	CO025	Colombia	N6.067217	W73.645411	MF097577	MF097700
*Lucilia*	*vulgata*	CO026	Colombia	N6.067217	W73.645411	MF097578	MF097701
*Lucilia*	*vulgata*	CO028	Colombia	N6.067217	W73.645411	MF097579	MF097702
Outgroups	**						
*Neobellieria*	*bullata*	BG64		As published^b^		JQ807156.1	–
*Ravinia*	*stimulans*	AZ60		As published^b^		JQ807112.1	–
*Sarcophaga*	*carnaria*	NICC0410		As published^c^		JQ582094.1	–
*Blaesoxipha*	*alcedo*	AY09		As published^b^		JQ806830.1	–
*Blaesoxipha*	*masculina*	AW36		As published^b^		JQ806832.1	–

**Notes.**

a Whitworth (2014).

b (Stamper et al., 2012, unpublished data).

c Jordaens et al. (2013b).

## Methods

### Specimens and DNA extraction

**Figure 1:**
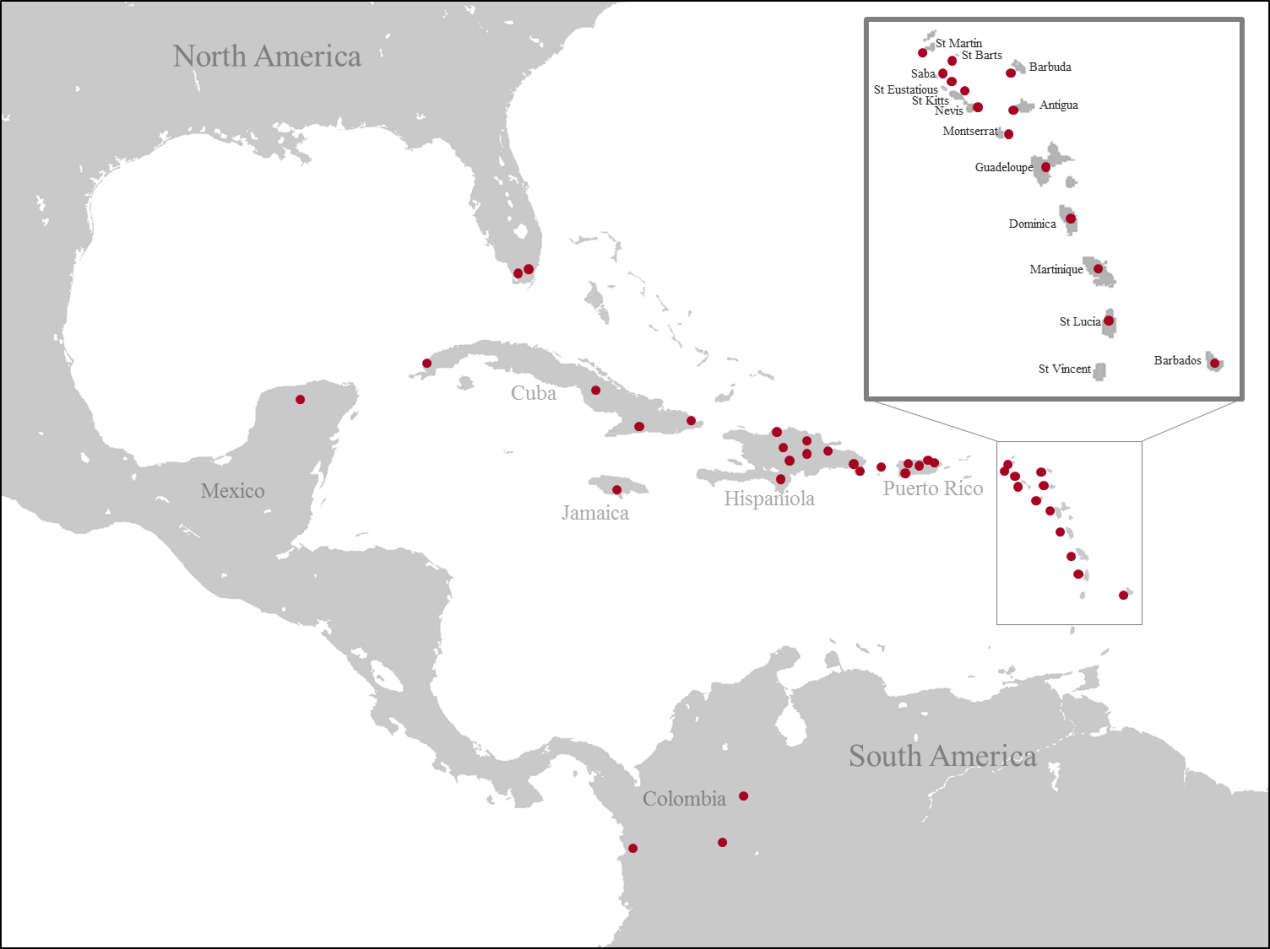
Map of collecting localities of all specimens used for the molecular analysis. (Image credit: https://commons.wikimedia.org/wiki/File:Caribbean_map_blank.png#filelinks).

A total of 473 specimens were included in this study. Of these, 468 represented ingroup taxa and five represented outgroup taxa from the family Sarcophagidae *(Sarcophaga Carnaria* Linnaeus, 1758; *Neobellieria bullata* Parker, 1916; *Ravinia stimulans* Walker, 1849; *Blaesoxipha masculina* Aldrich, 1916 and *Blaesoxipha alcedo* Aldrich, 1916). We used a total of 600 DNA sequences and we obtained 521 (COI  = 398, ITS2  = 123) while 79 (COI  = 44, ITS2  = 35) were previously published (Table 1). The specimens were collected throughout the Caribbean (Fig. 1) from between 2011 and 2013 (see Table 1 for details). All specimens were collected under appropriate permits: USA, Florida, Everglades, United States Department of the Interior National Park Service EVER-2013-SCI-0028; Puerto Rico, DRNA: 2011-IC-035 (O-VS-PVS15-SJ-00474-08042011); Jamaica, NEPA, reference number #18/27; USA, USDI National Park Service, EVER-2013-SCI-0028; Costa Rica, SINAC, pasaporte científico no. 05933, resolución no. 019-2013-SINAC; Cuba, Departamento de Recursos Naturales, PE 2012/05, 2012003 and 2012001; Dominican Republic, Ministerio de Medio Ambiente y Recursos Naturales, no 0577; Colombia, Authoridad Nacional de Licencias Ambientales, 18.497.666 issued to Alexander Gómez Mejía; Saba, The Executive Council of the Public Entity Saba, no 112/2013; Martinique, Ministère de L’Écologie, du Développement Durable, et de L‘Énergie; Nevis, Nevis Historical & Conservation Society, no F001; Barbados, Ministry of Environment and Drainage, no 8434∕56∕1 Vol. II. Although *L. vulgata*, *L. mexicana* and *L. coeruleiviridis* are not present in the Caribbean islands, they are included as outgroups to the Calliphoridae from the West Indies. James (1970) reported *L. coeruleiviridis* from Cuba, however, this is likely an error as no specimens have been seen in collections from the region (Whitworth, 2010) and no specimens were collected during this study. All specimens, except the ones from Mexico, were collected using a novel trap designed for this study. We modified a standard butterfly trap by adding a conic form on the top with a vessel attached to the highest point like in the Malaise trap. Flies entered the trap attracted by the bait (chicken) and funneled into the collecting vessel containing 95% ethanol. Traps were hung 1m off the ground and were used to collect flies for 2–3 days at each locality. These traps proved efficient in collecting specimens for our molecular purposes, given that caught specimens were preserved in ethanol while the trap remained in the field. Collected specimens were transferred to Whirl-paks with 95% ethanol and stored at −20 °C. Adults were identified using the Whitworth (2010) taxonomic keys and the specimens with uncertain identity were sent to Dr. Whitworth at Washington State University for detailed examination and species confirmation. DNA was isolated from thoracic muscle or two legs of each individual with the QIAGEN DNeasy Tissue Kit (Qiagen, Inc., Valencia, CA). The remainder of the specimen was retained as a voucher currently held by the Agnarsson Lab; they will be placed in the Zadock Thompson Zoological Collections at the UVM Natural History Museum following completion of other studies currently being conducted using the material.

### PCR amplification and sequencing

A region of the mitochondrial genome encoding COI was amplified in a single fragment using the primers LCO1490 (Folmer et al., 1994), and C1-N-2776 (Hedin & Maddison, 2001). Those primers amplified successfully in all Calliphoridae except *Lucilia* Robineau-Desvoidy. From the eight Caribbean species of *Lucilia*, only *Lucilia retroversa* amplified successfully using these primers. For the remaining *Lucilia* species two different primer-pairs were used. The Primer 1 (Gibson et al., 2011) with C1-N-2191 (Simon et al., 1994) and the C1-J-1751 (Gibson et al., 2011) with *C2-N-3014*. For the second internal transcribed spacer ITS2 we used the primers ITS4 and ITS5.8 (White et al., 1990). The primer sequences and protocols are listed in Table 2. Amplified fragments were sequenced in both directions by University of Arizona Genetics Core. Sequences were interpreted from chromatograms using Phred and Phrap (Green, 1999; Green & Ewing, 2002) using the Chromaseq module (Maddison & Maddison, 2010a) in Mesquite 3.03 (Maddison & Maddison, 2010b) with default parameters. The sequences were then proofread by examining chromatograms by eye. Alignments were done using MAFFT (Katoh et al., 2002) through the online portal EMBL-EBI with default settings. The matrices were exported to Mesquite 3.03 (Maddison & Maddison, 2010b) and the translation of coding sequences to proteins for COI were checked for potential errors.

**Table 2:** COI amplification primers and protocols.

Primer name		Sequence (5′–3′)	Protocol						Source protocol
			**ID**	**CY**	**D**	**AN**	**E**	**FE**	
LCO1490	F	GGTCAACAAATCATAAAGATATTGG	95 °C 2 min	35	95 °C 30 s	44 °C 45 s	72 °C 45 s	72 °C 10 min	Agnarsson, Maddison & Aviles (2007)
CI-N-2776	R	GGATAATCAGAATATCGTCGAGG							
Primer 1	F	TACAATTTATCGCCTAAACTTCAGCC	95 °C 3 min	35	94 °C 15 s	51 °C 15 s	72 °C 30 s	72 °C 5 min	DeBry et al. (2013)
C1-N-2191	R	CCCGGTAAAATTAAAATATAAACTTC							
C1-J-1751	F	GGAGCTCCTGACATAGCATTCCC	94 °C 90 s	36	94 °C 22 s	48 °C 30 s	72 °C 80 s	72 °C 60 s	Harvey et al. (2003)
C2-N-3014	R	TCCATTGCACTAATCTGCCATATTA							
ITS4	F	TCCTCCGCTTATTGATATGC	94 °C 2 min	38	94 °C 30 s	44 °C 35 s	72 °C 30 s	72 °C 3 min	Agnarsson (2010)
ITS5.8	R	GGGACGATGAAGAACGCAGC							

**Notes.**

F Forward R Reverse ID Initial denaturation CY cycles D Denaturation AN annealing E Extension FE Final extension

### Phylogenetic analysis

The COI gene was partitioned by codon positions, each partition and ITS2 gene were exported from Mesquite for model choice. The appropriate models were chosen using jModeltest v2.1.4 (Posada & Crandall, 1998), and the AIC criterion (Posada & Buckley, 2004). The corresponding model of evolution was used for the Bayesian analysis: GTR + Γ + I for COI1st, F81+ I for COI2nd, GTR + Γ for COI3rd and HKY + Γ + I for ITS2. We ran the MC^3^(Metropolis Coupled Markov Chain Monte Carlo) chain in MrBayes v3.2.3 (Huelsenbeck & Ronquist, 2001) through the online portal Cipres Science Gateway v3.3 (Miller, Pfeiffer & Schwartz, 2010). The analysis was run for 20,000,000 generations, sampling every 1,000 generations, and the sample points of the first 5,000,000 generations were discarded as ‘burnin’, after which the chains had reached stationarity as determined by analysis in Tracer (Rambaut & Drummond, 2009). Maximum likelihood (ML) analysis of the concatenated matrix was done in Garli (Zwickl, 2006) using the same partitioning scheme and models. Sequences were submitted to GenBank and BOLD.

### Species delimitation

We used MEGA6 to calculate genetic distances within and among species level clades suggested by the barcoding analysis of the COI data and by morphology. We used the species delimitation plugin in Geneious 8.1.5 (Kearse et al., 2012; Masters, Fan & Ross, 2011) to estimate species limits under Rosenberg’s reciprocal monophyly P(AB) (Rosenberg, 2007) and Rodrigo’s P(RD) method (Rodrigo et al., 2008). For this analysis we used a 317 taxa subset of our data, produced by reducing the most densely sampled species like *Co. minima, Co. macellaria, Ch. rufifacies* and *L. retroversa* to 38 exemplars since P(RD) probability cannot be computed when there are more than 40 exemplars per clade. We also estimated the probability of population identification of a hypothetical sample based on the groups being tested P ID (Strict) and P ID (Liberal). The genealogical sorting index (gsi) statistic (Cummings, Neel & Shaw, 2008) was calculated using the gsi webserver (http://genealogicalsorting.org) on the estimated tree. As genetic distances in MEGA6, gsi and species delimitation metrics from Geneious require a priory species designation, 26 putative species were assigned to the data based on combined analysis of phylogenetic topology from COI and morphological and geographic information. Finally, we used a single locus Bayesian implementation (bPTP) of the Poisson tree processes model (Zhang et al., 2013) to infer putative species boundaries on a given single locus phylogenetic input tree available on the webserver: http://species.h-its.org/ptp/. The analysis was run as a rooted tree from the MrBayes analysis, for 500,000 generations with 10% burnin removed. For gsi and bPTP analysis we reduced the data to 103 taxa representing the 26 putative species because of limitations of the server.

**Figure 2:**
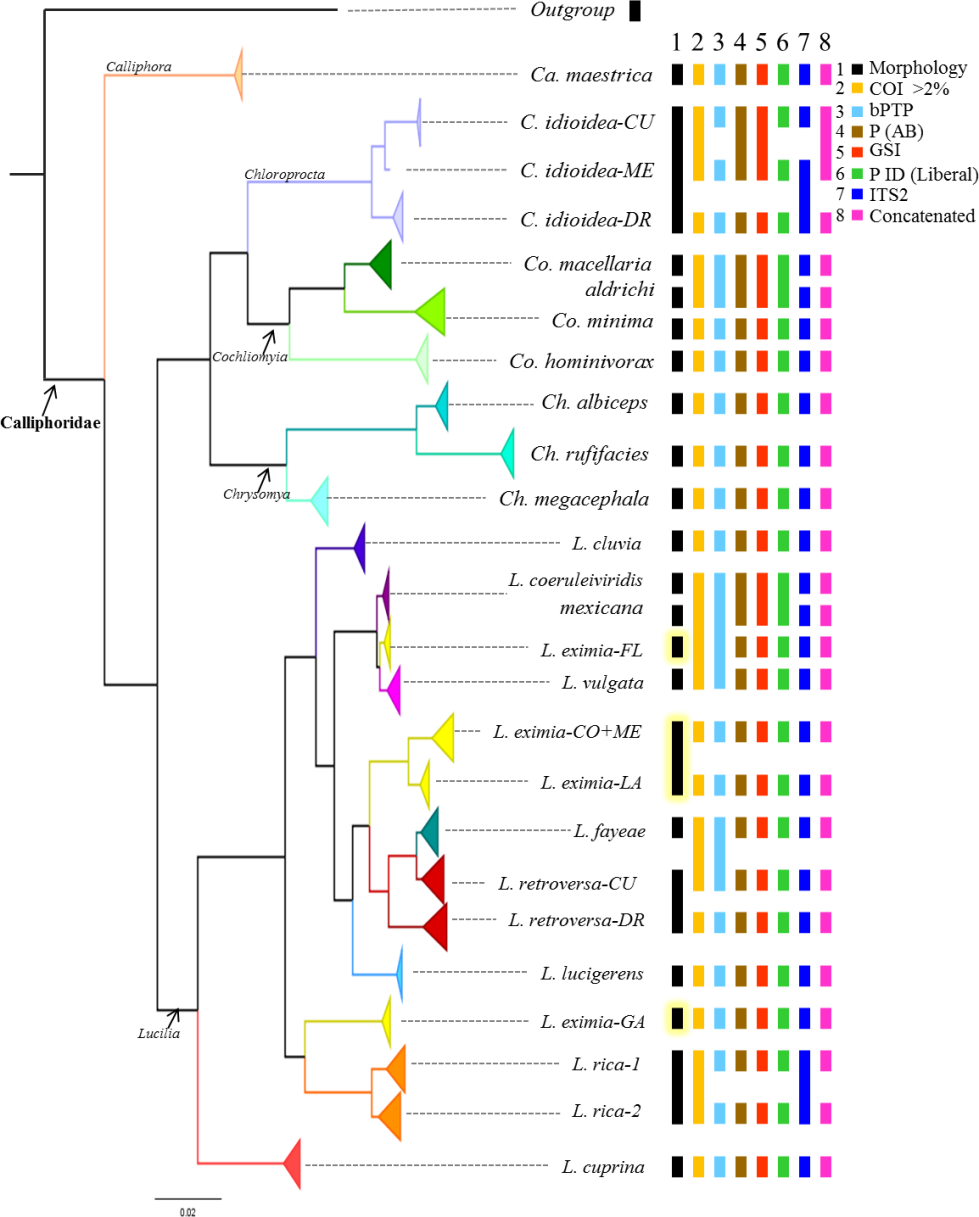
Summary of the Bayesian tree based on the COI dataset including 442 individuals, with the results of four different species delimitation approaches in addition to morphology, genetic distances of >2% mtDNA, ITS2 and the concatenated matrix. See Fig. S1 for bootstrap support values.

## Results

We present by far the most extensive DNA barcoding dataset of Calliphoridae from the Caribbean. It includes a ∼1,200 bp fragment of the mitochondrial COI gene from 437 Calliphoridae specimens and ∼450 bp of the ITS2 gene from 158 specimens chosen to represent unique COI haplotypes of all putative species and all localities (20 different islands in the Caribbean plus Florida, Colombia and Mexico). Ninety nine of the sequences are from specimens collected in the mainland and the other 496 are from the Caribbean Islands. In total, we included 19 species of Calliphoridae identified morphologically (Whitworth, 2006; Whitworth, 2010), 16 of them reported from the Caribbean and three species, *L. coeruleiviridis, L. mexicana* and *L. vulgata,* from the mainland. The sequences from the Caribbean represent 16 of the 18 species of forensically important Calliphoridae that occur in the West Indies plus one of the most important livestock pest parasites in the Americas, *C. hominivorax* (Whitworth, 2010). The two species not included in this dataset are reported from Bahamas (*Phormia regina*) and Trinidad (*Hemilucilia segmentaria*), where we were not able to sample. For most species we included numerous exemplars, covering the geographic range of each species in the region.

### Species delimitation using COI

Although based on traditional taxonomy we recognized 19 species of Calliphoridae in this study, COI gene analyses suggest that the diversity of Calliphoridae in the Caribbean is greater than morphology can detect. The phylogenetic analysis of COI recuperates 24 distinct clades (Fig. 2, Fig. S1), showing substantial geographic variation for *L. eximia* (four clades)*, C. idioidea* (three clades), *L. retroversa* (two clades) and *L. rica* (two clades). However, COI did not distinguish between the pairs, *Co. macellaria* and *Co. aldrichi* from the Caribbean and *L. coeruleiviridis* and *L. mexicana* from the mainland. These four species are clearly identifiable based on morphological characteristics. Most putative species lineages showed genetic distances >2.7% (Table 3) and most of them are separated by a barcoding gap (Table 4). All species delimitation methods supported *Ca. maestrica, C. idioidea-DR, Co. minima, Co. hominivorax, Ch. albiceps, Ch. rufifacies, Ch. megacephala, L. cluvia, L. cuprina, L. eximia-CO+ME, L. eximia-LA, Lucilia eximia-GA L. lucigerens, Lucilia retroversa-DR,* and *L. rica* 1 and 2 (Fig. 2, Table 5); however, the other eight putative species were poorly supported in our analyses. Lower divergences, between 0.5 and 1.2% were found between clades, *L. coeruleiviridis+L. mexicana*, *L. vulgata* and *L. eximia-FL*, *L. fayeae* and *L. retroversa CU*, and between *L. rica* 1 and 2 (Table 3). All but bPTP methods of species determination supported *L. eximia-FL* clade, *L. vulgata, L. fayeae, L. retroversa*-CU (Table 5). Regarding *C. idioidea*, the Cuban and Mexico species-clades are only supported by bPTP and P ID (liberal). The bPTP analysis estimated between 21 and 29 species including the initial 26 putative species. Other species delimitation methods showed similar results, 22 putative species had P ID (liberal) higher of 89, 20 had significant Rosenberg values and 21 had GSI values of 100. All species determination methods fail in distinguishing between the pairs *Co. macellaria* and *Co. aldrichi*, and *L. coeruleiviridis* and *L. mexicana* as sequence divergences between species pairs are extremely low <0.08%. Given that no one method can distinguish between these species, the addition of ITS2 as a second barcoding locus was necessary to clarify the monophyly and validity of these species and increase the confidence of delimitation and identification of species with low genetic divergences.

**Table 3:** Genetic distances expressed in percentage among the 26 putative species groups as determined by an analysis in MEGA6.

Putative species		1	2	3	4	5	6	7	8	9	10	11	12	13	14	15	16	17	18	19	20	21	22	23	24	25	26
1	*Ca. maestrica*																										
2	*C. idioidea-CU*	16.3																									
3	*C. idioidea-DR*	15.3	2.8																								
4	*C. idioidea-ME*	15.6	2.1	2.1																							
5	*Ch. albiceps*	15.5	13.8	13.1	13.1																						
6	*Ch. megacephala*	14.4	9.5	10.6	10.2	5.7																					
7	*Ch. rufifacies*	15.5	14.5	14.1	14.1	2.8	6.7																				
8	*Co. aldrichi*	14.4	10.6	9.5	9.9	10.2	9.2	12.0																			
9	*Co. hominivorax*	13.7	8.7	9.1	8.0	11.5	9.8	12.6	8.4																		
10	*Co. macellaria*	14.4	10.6	9.5	9.9	10.3	9.2	12.0	0.1	8.4																	
11	*Co. minima*	15.7	10.5	10.5	10.2	9.8	8.8	11.0	4.2	9.7	4.2																
12	*L.. cluvia*	11.1	11.4	12.1	12.1	14.9	11.4	14.5	12.4	11.6	12.4	13.7															
13	*L.. coeruleiviridis*	12.1	11.3	13.4	13.4	15.9	12.7	15.2	11.7	12.6	11.6	11.4	4.6														
14	*L. cuprina*	11.6	9.2	9.5	10.2	13.1	9.5	13.8	10.6	11.9	10.6	11.2	8.2	8.5													
15	*L. eximia-CO-ME*	12.4	12.3	11.7	12.4	14.0	11.8	14.0	12.8	13.0	12.8	13.4	5.4	7.1	7.6												
16	*L. vulgata*	11.4	11.3	12.7	12.7	15.9	12.7	15.9	11.7	12.6	11.7	12.0	3.9	0.7	8.5	6.4											
17	*L. eximia-FL*	11.6	11.0	12.4	12.4	15.5	12.4	14.8	12.0	12.4	11.9	11.1	4.8	1.2	8.7	6.9	1.2										
18	*L. eximia-GA*	13.5	12.0	13.4	14.1	14.5	12.7	15.2	12.7	13.7	12.7	13.0	7.1	4.9	9.5	7.4	4.9	5.5									
19	*L. eximia-LA*	12.1	11.3	9.9	11.3	13.1	11.3	13.8	11.7	11.5	11.6	12.3	4.3	6.0	6.7	2.6	5.3	5.8	6.4								
20	*L. fayeae*	13.2	11.2	12.6	12.6	13.5	11.7	13.9	12.6	10.9	12.6	13.3	4.7	4.9	8.4	5.7	4.9	5.4	5.6	4.5							
21	*L. lucigerens*	11.9	11.7	12.4	12.4	14.5	11.7	14.1	12.7	11.9	12.7	12.7	3.2	4.9	7.8	3.7	4.2	4.8	6.0	3.2	4.2						
22	*L. mexicana*	12.1	11.3	13.4	13.4	15.9	12.7	15.2	11.7	12.6	11.6	11.4	4.6	0.0	8.5	7.1	0.7	1.2	4.9	6.0	4.9	4.9					
23	*L. retroversa-CU*	13.7	11.2	12.6	12.6	14.0	12.2	14.4	12.6	11.4	12.6	13.3	5.2	4.8	8.4	5.6	4.8	5.4	5.3	4.5	0.5	4.1	4.8				
24	*L. retroversa-DR*	13.5	12.4	13.1	13.1	14.8	13.4	14.5	12.7	13.3	12.7	13.4	4.0	5.0	9.2	5.4	4.3	4.8	5.7	4.6	2.8	3.6	5.0	2.7			
25	*L. rica_1*	13.9	12.1	12.1	11.6	14.8	11.9	14.7	13.0	11.1	13.0	13.6	6.1	6.8	8.2	6.6	6.1	6.6	7.5	5.4	5.0	5.4	6.8	4.7	5.0		
26	*L. rica_2*	13.4	11.9	11.9	11.2	14.7	11.6	14.8	12.6	10.7	12.6	13.3	5.6	6.3	8.0	6.4	5.6	6.1	7.3	5.6	5.6	5.2	6.3	5.3	5.3	1.0	

**Notes.**

CO Colombia CU Cuba DR Dominican Republic FL Florida GA Greater Antilles LA Lesser Antilles ME Mexico

**Table 4:** Genetic distances within the 26 putative species groups, as determined by an analysis in MEGA6. The values are expressed as a percentage.

Putative species	% variation within species
*Ca. maestrica*	0.14
*C. idioidea-CU*	0.00
*C. idioidea-DR*	0.00
*C. idioidea-ME*	n/a
*Ch. albiceps*	0.00
*Ch. megacephala*	0.00
*Ch. rufifacies*	0.01
*Co. aldrichi*	0.00
*Co. hominivorax*	0.24
*Co. macellaria*	0.15
*Co. minima*	0.29
*L.. cluvia*	0.10
*L.. coeruleiviridis*	0.00
*L. cuprina*	0.00
*L. eximia-CO-ME*	0.61
*L. vulgata*	0.00
*L. eximia-FL*	1.06
*L. eximia-GA*	0.00
*L. eximia-LA*	0.00
*L. fayeae*	0.14
*L. lucigerens*	0.00
*L. mexicana*	0.00
*L. retroversa-CU*	0.18
*L. retroversa-DR*	0.08
*L. rica_1*	0.40
*L. rica_2*	0.15

**Notes.**

CO Colombia CU Cuba DR Dominican Republic FL Florida GA Greater Antilles LA Lesser Antilles ME Mexico

**Table 5:** Results of species delimitation analysis based on COI. The various measures of distance, isolation and exclusivity metrics of these clades follow including: (D), the probability of population identification of a hypothetical sample based on the groups being tested (P ID (Strict) and P ID (Liberal)), Rosenberg’s reciprocal monophyly (P(AB)), the genealogical sorting index (gsi), and a single locus Bayesian implementation of the Poisson tree processes model (bPTP). Sp congru. refers to species hypothesis that are congruent with all methods, and Sp cons. is our conservative estimate of actual species richness based on agreement among all methods and >2% mtDNA sequence divergence. Morph refers to species richness based morphology and Concat. refers to species richness based on the concatenated tree.

Putative species	Mono	D Intra	D Inter	Dtra/ Dter	P ID(Strict)	P ID(Liberal)	P(AB)	GSI	bPTP	Sp congru	Sp cons	Morph	Concat
1. *C. maestrica*	yes	0.001	0.096	0.01	0.93 (0.80, 1.0)	0.98 (0.88, 1.0)	NAN	1	Y	1	1	1	1
2*. C. idioidea-CU*	yes	0.0009	0.012	0.07	0.74 (0.57, 0.92)	0.97 (0.82, 1.0)	0.17	1	Y	2	2	2	2
3. *C. idioidea-ME*	yes	n/a	0.012	n/a	n/a	0.96 (0.83, 1.0)	0.17	NA	Y				
4. *C. idioidea-DR*	yes	0.003	0.014	0.19	0.81 (0.68, 0.93)	0.95 (0.85, 1.0)	1.98E −03	1	Y	3	3		3
5. *Co. aldrichi*	no	0.0008	0.002	0.46	0.82 (0.75, 0.89)	0.95 (0.91, 0.99)	NA	0.39	N	4	4	3	4
6. *Co. macellaria*	no	0.003	0.002	1.47	0.00 (0.00, 0.00)	0.31 (0.28, 0.34)	NA	0.61	N			4	5
7. *Co. minima*	yes	0.002	0.030	0.07	0.97 (0.92, 1.0)	0.99 (0.96, 1.0)	6.30E −27	1	Y	5	5	5	6
8. *Co. hominivorax*	yes	0.004	0.066	0.07	0.75 (0.57, 0.92)	0.97 (0.83, 1.0)	1.90E −07	1	Y	6	6	6	7
9. *Ch. albiceps*	yes	0.002	0.033	0.05	0.90 (0.77, 1.0)	0.97 (0.87, 1.0)	4.90E −08	1	Y	7	7	7	8
10. *Ch. rufifacies*	yes	0.0009	0.033	0.03	0.99 (0.93, 1.0)	1.00 (0.97, 1.0)	4.90E −08	1	Y	8	8	8	9
11. *Ch. megacephala*	yes	0.001	0.054	0.02	0.99 (0.94, 1.0)	1.00 (0.97, 1.0)	1.40E −24	1	Y	9	9	9	10
12. *L. cluvia*	yes	0.002	0.033	0.07	0.91 (0.81, 1.0)	0.98 (0.92, 1.0)	7.10E −12	1	Y	10	10	10	11
13. *L. coeruleiviridis*	no	0.0008	0.0008	1.12	0.18 (0.05, 0.31)	0.49 (0.38, 0.59)	NA	0.59	N	11	11	11	12
14. *L. mexicana*	no	0.0007	0.0008	0.88	0.20 (0.02, 0.39)	0.51 (0.36, 0.66)	NA	0.49	N			12	13
15. *L. eximia-FL*	yes	0.002	0.005	0.40	0.39 (0.24, 0.54)	0.74 (0.58, 0.89)	0.03	1	N			13	14
16. *L. vulgata*	yes	0.002	0.007	0.32	0.65 (0.51, 0.79)	0.89 (0.78, 1.0)	0.03	1	N			14	15
17. *L. eximia-ME-CO*	yes	0.004	0.016	0.27	0.82 (0.71, 0.92)	0.93 (0.87, 0.99)	3.60E −04	1	Y	12	12		16
18. *L. eximia-LA*	yes	0.002	0.016	0.12	0.79 (0.64, 0.93)	0.95 (0.84, 1.0)	3.60E −04	1	Y	13	13		
19. *L. fayeae*	yes	0.002	0.008	0.31	0.82 (0.73, 0.91)	0.94 (0.89, 0.99)	2.40E −06	1	N	14	14	15	17
20. *L. retroversa-CU*	yes	0.004	0.008	0.46	0.75 (0.67, 0.84)	0.92 (0.87, 0.97)	2.40E −06	1	N			16	18
21. *L. retroversa-DR*	yes	0.002	0.024	0.09	0.96 (0.91, 1.0)	0.99 (0.96, 1.0)	2.60E −14	1	Y	15	15		19
22. *L. lucigerens*	yes	0.002	0.035	0.05	0.76 (0.58, 0.94)	0.98 (0.84, 1.0)	9.90E −07	1	Y	16	16	17	20
23. *L. eximia-GA*	yes	0.001	0.048	0.03	0.98 (0.91, 1.0)	1.00 (0.96, 1.0)	1.30E −11	1	Y	17	17		21
24. *L. rica_1*	yes	0.003	0.011	0.24	0.90 (0.83, 0.96)	0.97 (0.92, 1.0)	4.40E −09	1	Y	18	18	18	22
25. *L. rica_2*	yes	0.002	0.011	0.22	0.90 (0.83, 0.97)	0.97 (0.92, 1.0)	4.40E −09	1	Y	19			23
26. *L. cuprina*	yes	0.002	0.076	0.03	0.98 (0.91, 1.0)	1.00 (0.96, 1.0)	4.30E −19	1	Y	20	19	19	24

**Notes.**

CO Colombia CU Cuba DR Dominican Republic FL Florida GA Greater Antilles LA Lesser Antilles ME Mexico

### Phylogenetic inference

**Figure 3:**
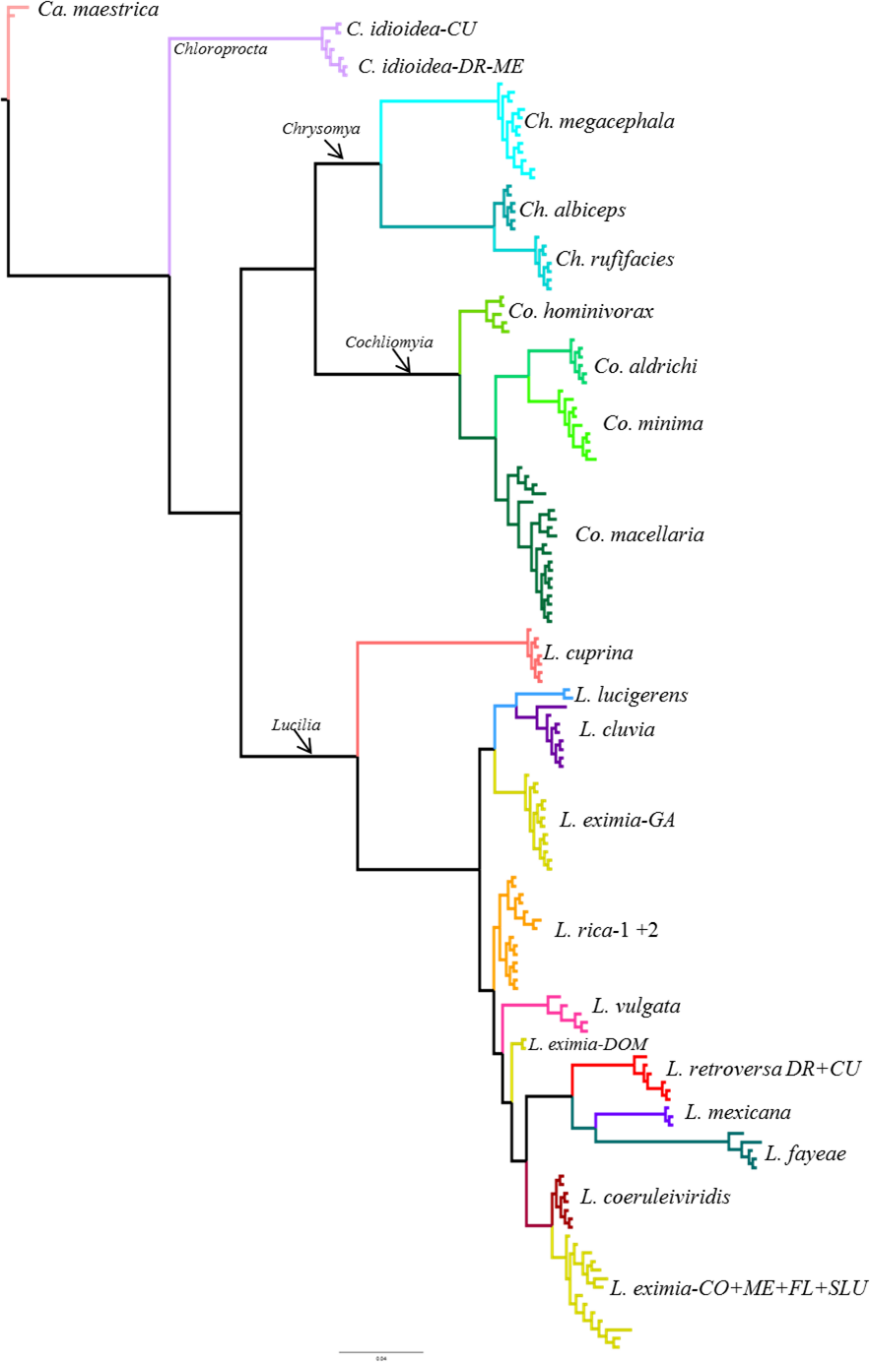
Bayesian tree based on ITS2 dataset including 158 specimens. Individual terminal taxa have been replaced with species names, while full taxon clade structure is retained. Colors represent different species based on morphology. See Fig. S2 for bootstrap support values.

**Figure 4:**
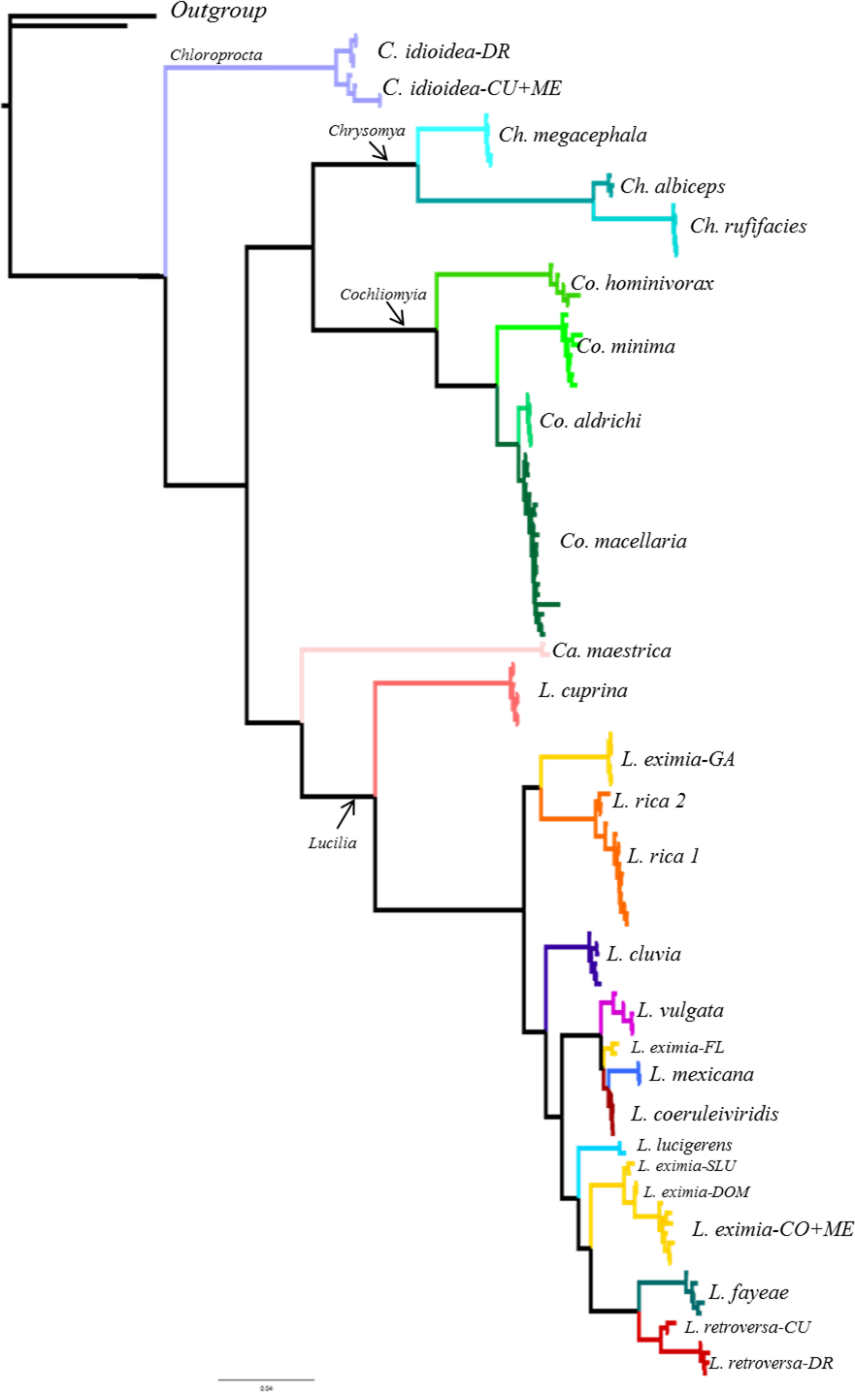
Bayesian tree based on the concatenate dataset including 137 specimens. Individual terminal taxa have been replaced with species names, while full taxon clade structure is retained. Colors represent different species based on morphology. See Fig. S3 for bootstrap support values.

From the 26 putative species analyzed here, 25 were represented by multiple individuals and one by a single individual in the COI analysis. All phylogenetic analyses (COI, ITS2, COI+ITS2) yielded well resolved trees with strong posterior probability support for most of the branches and broadly agreed on species limits but with some differences in topology (Figs. 2–4, Figs. S1–S3). The Bayesian analysis of the ITS2 supported the monophyly of 21 of 26 putative species. It recovered the monophyly of *Co. aldrichi, Co. macellaria, L. mexicana* and *L. coeruleiviridis,* which failed with all other analysis. However it did not recover the geographic variation of *C. idioidea* from Mexico and Dominican Republic, *L. retroversa* from Cuba and Dominican Republic or *L. rica* 1 and 2, and it only recovers three of the four *L. eximia* clades indicated by COI analyses (Fig. 3, Fig. S2). The concatenated tree supports 24 of the 26 putative species including two clades within *L. retroversa*, *L. rica*, and *C. idioidea*, and three clades within *L. eximia*. The concatenated matrix did not support the monophyly of *C. idioidea-CU* that is nested within *C. idioidea-ME* and *L. eximia-CO+ME* nested within *L. eximia-LA* (Fig. 4, Fig. S3).

## Discussion

Accurate identification of insects is a crucial step to using them as reliable evidence in legal investigations. Although morphology has been successfully used to identify immature specimens involved in cadaveric decomposition (Cardoso et al., 2014; Florez & Wolff, 2009; Szpila et al., 2013a; Szpila et al., 2013b; Szpila et al., 2014; Szpila & Villet, 2011; Wells, Byrd & Tantawi, 1999), this approach depends on the availability of taxonomic keys of the species present in the region. In the Caribbean, the immature stages of 11 species are unknown and other approaches are needed in order to identify them. Besides this, morphology may overlook potentially cryptic species and cannot be used on incomplete or destroyed specimens found on a crime scene. Here, we show DNA barcoding to be useful in overcoming these problems and provide tools to accelerate the identification and discovery of species. This is particularly important in areas like the Caribbean, where studies of insects involved in cadaveric decomposition are scarce (Whitworth, 2010; Yusseff-Vanegas & Agnarsson, 2016; Yusseff-Vanegas, 2007; Yusseff-Vanegas, 2014). One of the first steps required for this approach is creating a reliable DNA barcode database that can be used with confidence in order to identify unknown specimens found in death scenes investigation (DeBry et al., 2013; Harvey et al., 2003).

The success of DNA barcoding relies on the quality of the underlying database used to compare DNA sequences of new samples. A good database should contain DNA barcodes of expertly identified individuals, and preferably taxon sampling covering the distribution range of each species. Our study complies with both requirements and is the first thorough molecular study of Calliphoridae from the Caribbean. It includes a representative collection from all but two forensically relevant Calliphoridae from the region, and covers the whole geographic range of most of the investigated species (Table 1). All specimens in this study were carefully identified using traditional morphological taxonomy (Whitworth, 2006; Whitworth, 2010; Whitworth, 2014) and each individual was successfully allocated to one of the currently recognized calliphorid species, except for specimen CO027 that could only be identified to the genus level. Although morphological identification of specimens collected in this study corresponded to 19 previously reported species (Whitworth, 2010), our results based on molecular data indicate higher diversity. In all, 26 putative species lineages were identified, and in particular our results indicate that *Lucilia* and *Chloroprocta* are more diverse than suggested by current taxonomy. COI recovered substantial geographic variation for *C. idioidea, L. eximia, L. retroversa* and *L. rica* such that molecular data indicate up to eleven putative species lineages that cannot be, or at least have not been, recognized by morphology.

*Lucilia eximia* is considered a widespread species found from the southern United States through Central America to southern South America (Whitworth, 2014). Nevertheless, our molecular results show four distinct genetic clusters with an average inter-cluster divergence from 2.5 to 7.4% (Table 3). The clusters are geographically structured and three of them are widely separated (Fig. 2, Fig. S1). The first one is the Greater Antilles cluster (GA) that includes specimens from Puerto Rico, Mona Island and Dominican Republic, the second is a small cluster that includes specimens from Florida (FL), the third one contains specimens from Colombia and Mexico (CO-MEX), and the fourth contains specimens from the Lesser Antilles islands of Dominica and Saint Lucia (LA). Similar results were reported by Solano, Wolff & Castrol (2013) and Whitworth (2014) where widely separate clades of *L. eximia* were found using DNA barcodes. All species delimitation methods supported the uniqueness and genetic isolation of the four clades, each showing low intra-clade divergence (<1%, Table 4), and thus likely representing four distinct species. Although we found some morphological variation between *L. eximia* from the mainland and islands and among islands as previously reported (James, 1967; Whitworth, 2010; Woodley & Hilburn, 1994), detailed revision of those specimens by Dr. Whitworth from Washington State University concluded that there is not enough evidence to separate them as different morphological species, suggesting they may be morphologically cryptic species. Further studies on these populations will be necessary to establish their taxonomic status.

*Lucilia rica* was collected throughout the Lesser Antilles and is very abundant in most of the islands (personal observation). Although James (1970) listed this species from Puerto Rico, we did not find any specimens after very extensive collections on the island. Thus, we believe that *L. rica* is restricted to the Lesser Antilles and has not dispersed beyond Anguilla. Whitworth (2010) reported this species from Antigua, Bermuda, Guadalupe and St. Lucia; however, we found it in eight more islands (Table 1) and our data showed two geographic clusters (Figs. 2, 4; Figs. S1, S3). The first cluster (*L. rica-1*) contains specimens from St Martin, Saba, St. Eustatius, St. Kitts, Nevis and Martinique and the second one (*L. rica 2*) from Barbuda, Antigua, Montserrat and Guadeloupe. Although the genetic distance between clades is low (1%), it is much greater than the intra-clade divergences (<0.3%). While all species delimitation methods support the possibility of two different species (Tables 5 and 6), we did not find morphological evidence to support it. Nevertheless, given that this is the most abundant *Lucilia* species of the Lesser Antilles, additional studies on these populations are important to determine if the genetic difference is due to intraspecific variation or if they are cryptic species.

For Lucilia retroversa we find two geographic clusters, one from Cuba and one from the Dominican Republic with an average mtDNA distance of 2.5% (Table 3) and with low intra-clade divergence (<0.2%, Table 4). Whitworth (2010) reported some morphological differences between specimens from Bahamas (which share morphology with Cuban specimens) and the Dominican Republic, but after examination of male and female genitalia he concluded that those differences were intraspecific variation. However, he noticed that our *L. retroversa* specimens have a brown basicosta instead of white or yellow basicosta which is an important character used to separate *L. retroversa* from other species (see taxonomic key in Whitworth, 2010). Given that all of our species delimitation results support two possible cryptic species, we recommend further detailed molecular and morphological studies of these populations to determine if they merit the description of a separate species.

**Table 6:** Results of species delimitation analysis based on the concatenated tree. (D), the probability of population identification of a hypothetical sample based on the groups being tested (P ID (Strict) and P ID (Liberal)), Rosenberg’s reciprocal monophyly (P(AB)).

Putative species	Closest species	Mono	D Intra	D Inter	Dtra/ Dter	P ID(Strict)	P ID(Liberal)	P(AB)
*1. Ca. maestrica*	*L. cuprina*	yes	0.005	0.19	0.03	0.58 (0.43, 0.73)	0.97 (0.82, 1.0)	1.00E−05
*2. L. cluvia*	*L. coeruleiviridis*	yes	0.005	0.05	0.10	0.87 (0.74, 0.99)	0.97 (0.87, 1.0)	5.50E−09
*3 L. coeruleiviridis*	*L. eximia-FL*	yes	0.001	0.01	0.14	0.84 (0.72, 0.97)	0.96 (0.86, 1.0)	0.01
*4. L. mexicana*	*L. coeruleiviridis*	yes	0.0009	0.02	0.06	0.75 (0.58, 0.93)	0.97 (0.83, 1.0)	0.01
*5. L. eximia-FL*	*L. coeruleiviridis*	yes	0.005	0.01	0.48	0.34 (0.19, 0.50)	0.69 (0.53, 0.84)	4.94E−03
*6. L. vulgata*	*L. coeruleiviridis*	yes	0.003	0.02	0.18	0.75 (0.60, 0.89)	0.94 (0.83, 1.0)	0.1
*7. L. eximiaCO-ME*	*L. eximia-LA*	yes	0.006	0.02	0.32	0.79 (0.69, 0.90)	0.92 (0.86, 0.99)	0.01
*8. L. fayeae*	*L. retroversa-CU*	yes	0.006	0.04	0.15	0.84 (0.71, 0.96)	0.96 (0.86, 1.0)	4.30E−04
*9. L. retroversa-CU*	*L. retroversa-DR*	yes	0.004	0.02	0.18	0.50 (0.35, 0.65)	0.87 (0.72, 1.0)	0.03
*10. L. lucigerens*	*L. eximia-LA*	yes	0.003	0.04	0.08	0.55 (0.40, 0.70)	0.93 (0.78, 1.0)	3.10E−04
*11 L. eximia-GA*	*L. rica 2*	yes	0.002	0.06	0.04	0.91 (0.78, 1.0)	0.98 (0.87, 1.0)	2.70E−06
*12. L. rica 1*	*L. rica 2*	yes	0.005	0.02	0.33	0.81 (0.72, 0.90)	0.94 (0.88, 0.99)	4.20E−04
*13. L. rica 2*	*L. rica 1*	yes	0.004	0.02	0.30	0.59 (0.42, 0.77)	0.84 (0.69, 0.98)	4.20E−04
*14. L. cuprina*	*L. cluvia*	yes	0.003	0.15	0.02	0.94 (0.83, 1.0)	1.00 (0.94, 1.0)	1.90E−11
*15. Ch. albiceps*	*Ch. rufifacies*	yes	0.003	0.04	0.06	0.75 (0.57, 0.93)	0.97 (0.83, 1.0)	2.98E−03
*16. Ch. rufifacies*	*Ch. albiceps*	yes	0.002	0.04	0.05	0.90 (0.78, 1.0)	0.97 (0.87, 1.0)	2.98E−03
*17. Ch. megacephala*	*Ch. albiceps*	yes	0.003	0.11	0.02	0.92 (0.79, 1.0)	0.98 (0.87, 1.0)	2.80E−05
*18. Co. aldrichi*	*Co. macellaria*	yes	0.002	0.01	0.13	0.85 (0.72, 0.97)	0.96 (0.86, 1.0)	4.70E−07
*19. Co. macellaria*	*Co. aldrichi*	yes	0.007	0.01	0.52	0.84 (0.78, 0.89)	0.96 (0.93, 0.99)	4.70E−07
*20. Co. minima*	*Co. aldrichi*	yes	0.007	0.05	0.14	0.88 (0.77, 0.99)	0.96 (0.90, 1.0)	4.50E−09
*21. Co. hominivorax*	*Co. aldrichi*	yes	0.007	0.09	0.08	0.88 (0.76, 1.0)	0.97 (0.87, 1.0)	1.00E−07
*22. C. idioidea-DR*	*C. idioidea-ME*	yes	0.003	0.02	0.19	0.74 (0.60, 0.88)	0.94 (0.83, 1.0)	4.08E−03
*23 C. idioidea-CU*	*C. idioidea-ME*	yes	0.001	0.01	0.06	0.56 (0.41, 0.71)	0.94 (0.79, 1.0)	0.33
*24: L. retroversa-DR*	*L. retroversa-CU*	yes	0.004	0.02	0.16	0.76 (0.62, 0.90)	0.94 (0.83, 1.0)	0.03
*25: C. idioidea-ME*	*C. idioidea-CU*	no	0.005	0.01	0.38	0.40 (0.24, 0.55)	0.75 (0.59, 0.90)	NA
*26. L. eximia-LA*	*L. eximiaCO-ME*	no	0.007	0.02	0.36	0.63 (0.48, 0.77)	0.88 (0.77, 0.99)	NA

**Notes.**

CO Colombia CU Cuba DR Dominican Republic FL Florida GA Greater Antilles LA Lesser Antilles ME Mexico

*Chloroprocta idioidea*, the only species of *Chloroprocta*, is a widespread species found from southern North America to southern South America (Dear, 1985; Whitworth, 2010). Our results show that *C. idioidea* is also geographically structured into three clades: one from Dominican Republic, one from Cuba and one from Mexico (Fig. 2, Fig. S1). Analysis of the genetic divergence between clades show more than 2% divergence between the Cuba and Dominican Republic clades but less than 2% divergence between the Mexico and Cuba clades (Table 3). Some authors (Hall, 1948; Shannon, 1926) believed there were two species of the genus in the Americas, however (Dear, 1985) concluded that there was only one single widespread species that exhibits some color variations which is dependent upon geographic distribution. Our molecular results indicate at least two, and perhaps three, separate species of *Chloroprocta*. All species delimitation methods (Table 5) and the concatenated matrix (Fig. 4, Fig. S3) suggest that the Dominican Republic versus the Cuba and Mexico clade are separate species, but were ambiguous about the status of *C. idioidea-CU* that is nested within *C. idioidea-ME*. Cuban and Mexican specimens are morphologically similar, dark-bluish in color with brownish to orange legs, however, as reported by Dear (1985) the Cuban females have brownish, instead of yellow-white calypters. Our specimens from Dominican Republic are similar to the southern USA specimens described by Dear (1985) but have darker post spiracles and clear wings with only the costa faintly tinted. Although we could see morphological differences between populations, those differences were based on a limited number of specimens (e.g., five specimens from Dominican Republic and three from Mexico). Further studies with larger number of specimens of *C. idioidea,* including detailed morphological descriptions and expanded molecular analysis, are necessary to further test species limits within this genus.

Our focus here is not to fully resolve calliphorid taxonomy. However, it is important to highlight the consequences of our findings for forensic entomology studies. Currently *L. eximia* is one of the most widespread and abundant *Lucilia* in the Neotropics (Whitworth, 2014). However, our results suggest that, in fact, this is not one widely distributed species, but potentially several species that differ in geographic range and possibly in biological traits (rates of development, diapause, habitat preference, feeding habits etc.). The same is true for *L. retroversa* and *C. idioidea*, both have genetically distinct clades in the Dominican Republic and in Cuba (Figs. 2 and 4). This finding will have direct consequences for the use of these species in legal investigations, if that variation reflects differences in behavior and biology, that can affect post mortem interval estimations (Tarone, Singh & Picard, 2015). Previous studies of *Phormia regina* (Byrd & Allen, 2001), *C. macellaria* and *C. rufifacies* (Yusseff-Vanegas, 2007) have shown that their developmental rate differ from different populations. Picard & Wells (2009) suggested that that variation is in part due to differences in population genetic structure, and for that reason, ecological data obtained from one population should not be generalized or extrapolated to other populations (Byrne et al., 1995). This is important at least for specimens collected in Cuba where both populations are present, probably as the result of recent dispersal of *L. retroversa* and *C. idoidea* from the Dominican Republic to Cuba. Our results (S1) show that two of the southeast Cuban specimens, CU007 (*L. retroversa*) and CU008 (*C. idioidea*), collected in Turquino National Park in Cuba (Table 1), cluster tightly with Dominican Republic specimens (S1). To confirm the genetic affinity of these specimens we added three more nuclear genes for a limited number of individuals from both populations and re-ran the analysis. The multi-gene analysis again strongly clustered CU007 and CU008 with the Dominican Republic specimens for each species. Thus, both the Dominican Republic and Cuban populations are clearly present in Cuba.

COI recuperated substantial geographic variation with high COI sequences divergence between populations of *Lucilia eximia*, *L. retroversa*, *L. rica* and *C. idioidea* (Fig. 2, Fig. S1), suggesting the possibility of different species (Hebert et al., 2003; Hebert, Ratnasingham & deWaard, 2003). However, genetic variation is not always indicative of species differentiation. For instance, studies including *Phormia regina* have found that the genetic distance between N American and W European populations is higher than 4% (Boehme, Amendt & Zehner, 2012; Desmyter & Gosselin, 2009). But after detailed molecular and morphological analysis of both populations, Jordaens et al. (2013a) concluded that the high differentiation at COI, COII and cyt*b*, but low (16S, nDNA) and lack of morphological differentiation, was indicative of substantial intraspecific mtDNA sequence divergence, rather than a species level differentiation. In light of those results, definite conclusions cannot yet be drawn regarding the taxonomy of these species. Further population level studies of the four species in question are therefore necessary. A comprehensive molecular analysis including several mitochondrial and nuclear genes in combination with morphological examination and detailed description of the genitalia, are required to determine if they are in fact different species, or if the genetic difference between populations is the product of intraspecific variation. Meanwhile the use of these species for forensic purposes should be evaluated carefully and with reference to genetic and behavioral differences among its populations.

Regarding the other Calliphoridae species, *Ca. maestrica, Co. minima, Co hominivorax, Ch. albiceps, Ch. rufifacies, Ch. megacephala, L. cluvia, L. cuprina* and *L. lucigerens*, all showed reciprocal monophyly with strong posterior probability support and all can be successfully identified using the DNA barcoding approach. All species delimitation methods, phylogenetic analysis of ITS2, and the concatenated tree support their monophyly and species status, and the results are congruent with morphology. *Calliphora maestrica* is the only *Calliphora* species reported for the Caribbean and is endemic from the region. This species was originally described from Sierra Maestra region in Cuba (Peris et al., 1998) and later reported also from Jamaica and Dominican Republic (Whitworth, 2010). Although we collected on all three islands, we only found *C. maestrica* in Villa Pajon, Dominican Republic, a cold region at altitudes >2,140 m. We did not find it in Cuba or Jamaica, likely due to lack of sampling at altitudes above 1,200 m on both islands.

The three species of *Chrysomya* were recently introduced to the New World (Baumgartner & Greenberg, 1984). Although Whitworth (2010) reported *Ch. megacephala* and *Ch. rufifacies* from Dominica, Dominican Republic, Jamaica and Puerto Rico, they are abundantly present in most of the islands being found from Cuba to Martinique (Table 1). In contrast, *Chrysomya albiceps* has more restricted distribution being found in islands closer to South America (Table 1, Whitworth, 2010). Although Dear (1985) reported this species from Puerto Rico, we did not find it after extensive collections on the island. That report was based on a single larva found in a goat, probably of *Ch. albiceps* but the species was not confirmed (Gagne, 1981). We believe that *Ch. albiceps* has not dispersed beyond Dominica and that the species reported by Dear (1985) was in fact *Ch. rufifacies*. Given the high dispersal abilities of the species of this genus (Baumgartner & Greenberg, 1984) and their invasive behavior (Aguiar-Coelho & Milward-De-Azevedo, 1998; De Andrade et al., 2002; Faria et al., 1999; Wells & Greenberg, 1992), it is not surprising to find them widely distributed and very well established throughout the Caribbean. They do not show any geographic structure, suggesting their recent colonization from the mainland and the constant gene flow among populations.

*Lucilia cluvia* and *L. cuprina*, are widely distributed flies found in different parts of the world (Byrd & Castner, 2010). *Lucilia cluvia* is considered rare (Whitworth, 2010). Although it has been reported from several locations in Puerto Rico, Cuba, and Martinique, we have only found two specimens in a suburban area in Toa Baja, Puerto Rico. *Lucilia cuprina* is reported from several islands in the Caribbean, but we only found it in urban areas of Puerto Rico as our focus on other islands was in non-urban areas. Finally *L. lucigerens* is an endemic species from Jamaica and was collected abundantly throughout the island.

DNA barcoding in animals typically employs a single mitochondrial marker for identification and delimitation of species (Hebert et al., 2003; Hebert, Ratnasingham & DeWaard, 2003), and this approach has shown to be useful in Calliphoridae species identification. However it does not reliably distinguish among some recently diverged species (Harvey et al., 2003; Nelson, Wallman & Dowton, 2007), leading to doubt that COI alone is sufficient for identification of species (Nelson, Wallman & Dowton, 2007; Wells, Wall & Stevens, 2007). Rather, the use of multiple markers has been suggested as a means to increase the accuracy of species identification. Indeed, our results show that COI barcoding successfully identified most species, but did not distinguish between the pairs *L. mexicana* and *L. coeruleiviridis* as previously reported (DeBry et al., 2013; Whitworth, 2014; Williams, Lamb & Villet, 2016) and between *Co. aldrichi* and *Co. macellaria* (Tables 3 and 5, Fig. S1 ). The latter species is considered one of the most important Calliphoridae for forensic studies in the Americas (see discussion in Yusseff-Vanegas & Agnarsson, 2016). Additionally, COI showed very low genetic divergences (<0.7%, Table 3) between the putative species *L. vulgata* and *L. coeruleiviridis*, and *L. fayeae* and *L. retroversa-CU*; species that are clearly distinguished based on morphological characteristics. This low genetic divergence may reflect short histories of reproductive isolation (Hebert, Ratnasingham & DeWaard, 2003), or mitochondrial introgression. In either case the addition of the nuclear gene ITS2 resolved the monophyly of the four species that COI alone did not support, and added resolution for uncertain groups with mtDNA genetic distances lower than 2%. These findings agreed with previous studies where the analysis of ITS2 resolved complex species delimitation (GilArriortua et al., 2014; Song, Wang & Liang, 2008), however, not always addition of more genes resolved the monophyly of the sister species like the case of *L. illustris* and *L. caesar*, where, after analysis including six genes, the monophyly remain unresolved (Sonet et al., 2012).

In sum, our study demonstrates the importance employing a second nuclear marker for barcoding analyses and species delimitation of calliphorids and the power of molecular data in combination with a complete reference database to enable identification of taxonomically and geographically diverse insects of forensic importance. The combination of the two markers supported the higher diversity of Calliphoridae in the Caribbean recovering the monophyly of nine of the eleven possible cryptic species. However, definite conclusion about the taxonomy of these species will depend on further studies combining molecular and morphological approaches.

## Conclusion

From almost a decade many studies have applied DNA-based methods for the identification of insects of forensic importance to enable identification of unknown insect specimens found in death scene investigations. However, this technique is not being implemented and the traditional time consuming methods of raising immature stages to adulthood is still in practice. The use of this approach has been unsuccessful because of lack of confidence due to sequence gaps and errors, unauthenticated reference DNA sequences in the database, and incomplete reference data set with partial taxon sampling. Thus, the base science foundation for application of DNA sequences analysis is unsolid for identification of evidentiary samples. Despite all studies of DNA based identification for insects involved in forensics, only a few of them include a complete reference data set. But even with a complete reference database, COI has failed in demonstrating reciprocal monophyly for several recently diverged species creating uncertainty about its use for identification. The addition of ITS2 as a second marker may be the key to increase certainty in identification and make this technique useful for forensic purposes. A great effort to build complete reference databases including extensive collections, accurate identification, geographical genetic variation for each targeted insect group and the addition of ITS2 as a second marker is needed. In general, COI barcodes are highly useful for species identification of the Caribbean calliphorids. ITS2 appears to be a good second marker that allows higher resolution and accurate identification of specimens that cannot be separated by COI alone. Our study provides, for the first time, a reliable dataset to accurately identify species of the family Calliphoridae from the Caribbean, and opens the door for future studies on biodiversity, biogeography, distribution and ecology of these forensically important flies.

##  Supplemental Information

10.7717/peerj.3516/supp-1 Figure S1 Phylogenetic relationship within Calliphoridae based on a Bayesian analysis of nucleotide data from COI Numbers indicate posterior probability support values. Specimen voucher codes referred to in Table 1 are shown following species names. For specimens from Lesser Antilles (LA), the three capital letters before the voucher code refers to the name of the islands abbreviated a follows: SBA, St. Barthelemy; SAB, Saba; BAR, Barbuda; NEV, Nevis; KIT, St. Kitts; MTQ, Martinique; ANT, Antigua; GUA, Guadeloupe; MON, Montserrat; EUS, St. Eustatius; SMA, St. Martin, SLU, St. Lucia; BBD Barbados.

10.7717/peerj.3516/supp-2 Figure S2 Phylogenetic relationship within Calliphoridae based on a Bayesian analysis of nucleotide data from ITS Numbers indicate posterior probability support values. Specimen voucher codes referred to in Table 1 are shown following species names. For specimens from Lesser Antilles (LA), the three capital letters before the voucher code refers to the name of the islands abbreviated a follows: SBA, St. Barthelemy; SAB, Saba; BAR, Barbuda; NEV, Nevis; KIT, St. Kitts; MTQ, Martinique; ANT, Antigua; GUA, Guadeloupe; MON, Montserrat; EUS, St. Eustatius; SMA, St. Martin, SLU, St. Lucia; BBD Barbados.

10.7717/peerj.3516/supp-3 Figure S3 Phylogenetic relationship within Calliphoridae based on based on partitioned Bayesian analysis of the combined gene (COI and ITS2) data set Numbers indicate posterior probability support values. Specimen voucher codes referred to in Table 1 are shown following species names. For specimens from Lesser Antilles (LA), the three capital letters before the voucher code refers to the name of the islands abbreviated a follows: SBA, St. Barthelemy; SAB, Saba; BAR, Barbuda; NEV, Nevis; KIT, St. Kitts; MTQ, Martinique; ANT, Antigua; GUA, Guadeloupe; MON, Montserrat; EUS, St. Eustatius; SMA, St. Martin, SLU, St. Lucia; BBD Barbados.

10.7717/peerj.3516/supp-4 Data S1 Data Matrix COI

10.7717/peerj.3516/supp-5 Data S2 Data Matrix ITS2

## Additional Information and Declarations

### Field Study Permissions

The following information was supplied relating to field study approvals (i.e., approving body and any reference numbers): All specimens were collected under appropriate permits: USA, Florida, Everglades, United States Department of the Interior National Park Service EVER-2013-SCI-0028; Puerto Rico, DRNA: 2011-IC-035 (O-VS-PVS15-SJ-00474-08042011); Jamaica, NEPA, reference number #18/27; USA, USDI National Park Service, EVER-2013-SCI-0028; Costa Rica, SINAC, pasaporte científico no. 05933, resolución no. 019-2013-SINAC; Cuba, Departamento de Recursos Naturales, PE 2012/05, 2012003 and 2012001; Dominican Republic, Ministerio de Medio Ambiente y Recursos Naturales, no 0577, Mexico, SEMARNAT scientific collector permit FAUT-0175 issued to Dr. Oscar Federico Francke Ballve, Oficio no. SGPA/DGVS/10102/13; Colombia, Authoridad Nacional de Licencias Ambientales, 18.497.666 issued to Alexander Gómez Mejía; Saba, The Executive Council of the Public Entity Saba, no 112/2013; Martinique, Ministère de L’Écologie, du Développement Durable, et de L‘Énergie; Nevis, Nevis Historical & Conservation Society, no F001; Barbados, Ministry of Environment and Drainage, no 8434/56/1 Vol. II.

### DNA Deposition

The following information was supplied regarding the deposition of DNA sequences: The GenBank accession numbers are in Table 1.

### Data Availability

The following information was supplied regarding data availability: The raw data has been supplied as a Supplementary File.

## References

[ref-1] Agnarsson I (2010). The utility of ITS2 in spider phylogenetics: notes on prior work and an example from *Anelosimus*. Journal of Arachnology.

[ref-2] Agnarsson I, Maddison WP, Aviles L (2007). The phylogeny of the social *Anelosimus* spiders (Araneae: Theridiidae) inferred from six molecular loci and morphology. Molecular Phylogenetics and Evolution.

[ref-3] Aguiar-Coelho VM, Milward-De-Azevedo EMV (1998). Combined rearing of *Cochliomyia macellaria* (Fabr.), *Chrysomya megacephala* (Fabr.) and *Chrysomya albiceps* (Wied.) (Dipt, Calliphoridae) under laboratory conditions. Journal of Applied Entomology-Zeitschrift Fur Angewandte Entomologie.

[ref-4] Aly SM, Wen JF (2013). Applicability of partial characterization of cytochrome oxidase I in identification of forensically important flies (Diptera) from China and Egypt. Parasitology Research.

[ref-5] Anslan S, Tedersoo L (2015). Performance of cytochrome c oxidase subunit I (COI), ribosomal DNA Large Subunit (LSU) and Internal Transcribed Spacer 2 (ITS2) in DNA barcoding of Collembola. European Journal of Soil Biology.

[ref-6] Baumgartner DL, Greenberg B (1984). The genus *Chrysomya* (diptera, calliphoridae) in the new world. Journal of Medical Entomology.

[ref-7] Boehme P, Amendt J, Zehner R (2012). The use of COI barcodes for molecular identification of forensically important fly species in Germany. Parasitology Research.

[ref-8] Byrd JH, Allen JC (2001). The development of the black blow fly, Phormia regina (Meigen). Forensic Science International.

[ref-9] Byrd JH, Castner JL (2010). Forensic entomology: the utility of arthropods in legal investigations.

[ref-10] Byrne AL, Camann MA, Cyr TL, Catts EP, Espelie KE (1995). Forensic implications of biochemical differences among geographic populations of the black blow fly, Phormia regina (meigen). Journal of Forensic Sciences.

[ref-11] Candek K, Kuntner M (2015). DNA barcoding gap: reliable species identification over morphological and geographical scales. Molecular Ecology Resources.

[ref-12] Cao XW, Liu J, Chen J, Zheng G, Kuntner M, Agnarsson I (2016). Rapid dissemination of taxonomic discoveries based on DNA barcoding and morphology. Scientific Reports.

[ref-13] Cardoso GA, Matiolli CC, De Azeredo-Espin AML, Torres TT (2014). Selection and validation of reference genes for functional studies in the Calliphoridae family. Journal of Insect Science.

[ref-14] Catts EP (1992). Problems in estimating the postmortem interval in death investigations. Journal of Agricultural Entomology.

[ref-15] Catts EP, Haskell NH (1990). Entomology and death: a procedural guide.

[ref-16] Chen CD, Nazni WA, Lee HL, Hashim R, Abdullah NA, Ramli R, Lau KW, Heo CC, Goh TG, Izzul AA, Sofian-Azirun M (2014). A preliminary report on ants (Hymenoptera: Formicidae) recovered from forensic entomological studies conducted in different ecological habitats in Malaysia. Tropical Biomedicine.

[ref-17] Chen Q, Bai J, Liu L, Lin H-B, Tang H, Zhao W, Zhou H-Z, Yan J-W, Liu Y-C, Song-Nian H (2009). Sequential analysis of mitochondrial COI gene for seven common sarcosaphagous flies (Diptera) in Beijing and the establishment of their DNA barcodes. Acta Entomologica Sinica.

[ref-18] Coddington JA, Agnarsson I, Cheng RC, Candek K, Driskell A, Frick H, Gregoric M, Kostanjsek R, Kropf C, Kweskin M, Lokovsek T, Pipan M, Vidergar N, Kuntner M (2016). DNA barcode data accurately assign higher spider taxa. Peerj.

[ref-19] Cummings MP, Neel MC, Shaw KL (2008). A genealogical approach to quantifying lineage divergence. Evolution.

[ref-20] De Andrade JB, Rocha FA, Rodrigues P, Rosa GS, Faria LD, Von Zuben CJ, Rossi MN, Godoy WAC (2002). Larval dispersal and predation in experimental populations of *Chrysomya albiceps* and *Cochliomyia macellaria* (Diptera: Calliphoridae). Memorias Do Instituto Oswaldo Cruz.

[ref-21] Dear JP (1985). A revision of the New World Chrysomyini (Diptera: Calliphoridae). Revista Brasileira De Zoologia.

[ref-22] DeBry RW, Timm A, Wong ES, Stamper T, Cookman C, Dahlem GA (2013). DNA-based identification of forensically important *Lucilia* (Diptera: Calliphoridae) in the Continental United States. Journal of Forensic Sciences.

[ref-23] Desmyter S, Gosselin M (2009). COI sequence variability between Chrysomyinae of forensic interest. Forensic Science International-Genetics.

[ref-24] Faria LDB, Orsi L, Trinca LA, Godoy WAC (1999). Larval predation by *Chrysomya albiceps* on *Cochliomyia macellaria*, *Chrysomya megacephala* and *Chrysomya putoria*. Entomologia Experimentalis Et Applicata.

[ref-25] Florez E, Wolff M (2009). Description and key to the main species of Calliphoridae (Diptera) larvae of forensic importance from Colombia. Neotropical Entomology.

[ref-26] Folmer O, Black M, Hoeh W, Lutz R, Vrijenhoek R (1994). DNA primers for amplification of mitochondrial cytochrome c oxidase subunit I from diverse metazoan invertebrates. Molecular Marine Biology and Biotechnology.

[ref-27] Gagne RJ (1981). *Chrysomya* spp, old world blow flies (Diptera: Calliphoridae) recently established in the Americas. Bulletin of the Entomological Society of America.

[ref-28] Gibson JF, Kelso S, Jackson MD, Kits JH, Miranda GFG, Skevington JH (2011). Diptera-specific polymerase chain reaction amplification primers of use in molecular phylogenetic research. Annals of the Entomological Society of America.

[ref-29] GilArriortua M, Bordas MIS, Kohnemann S, Pfeiffer H, De Pancorbo MM (2014). Molecular differentiation of Central European blowfly species (Diptera, Calliphoridae) using mitochondrial and nuclear genetic markers. Forensic Science International.

[ref-30] Goff ML (2000). A fly for the prosecution: how insect evidence helps solve crimes.

[ref-31] Green P (1999). http://phraporg.

[ref-32] Green P, Ewing B (2002). http://phraporg.

[ref-33] Greenberg B, Szyska ML (1984). Immature stages and biology of 15 species of peruvian calliphoridae (Diptera). Annals of the Entomological Society of America.

[ref-34] Hall DG (1948). The blowflies of North America.

[ref-35] Harvey ML, Gaudieri S, Villet MH, Dadour IR (2008). A global study of forensically significant calliphorids: implications for identification. Forensic Science International.

[ref-36] Harvey ML, Mansell MW, Villet MH, Dadour IR (2003). Molecular identification of some forensically important blowflies of southern Africa and Australia. Medical and Veterinary Entomology.

[ref-37] Hebert PDN, Cywinska A, Ball SL, DeWaard JR (2003). Biological identifications through DNA barcodes. Proceedings of the Royal Society B-Biological Sciences.

[ref-38] Hebert PDN, Penton EH, Burns JM, Janzen DH, Hallwachs W (2004a). Ten species in one: DNA barcoding reveals cryptic species in the neotropical skipper butterfly *Astraptes fulgerator*. Proceedings of the National Academy of Sciences of the United States of America.

[ref-39] Hebert PDN, Ratnasingham S, DeWaard JR (2003). Barcoding animal life: cytochrome c oxidase subunit 1 divergences among closely related species. Proceedings of the Royal Society B-Biological Sciences.

[ref-40] Hebert PDN, Stoeckle MY, Zemlak TS, Francis CM (2004b). Identification of birds through DNA barcodes. PLOS Biology.

[ref-41] Hedin MC, Maddison WP (2001). A combined molecular approach to phylogeny of the lumping spider subfamily Dendryphantinae (Araneae : Salticidae). Molecular Phylogenetics and Evolution.

[ref-42] Huelsenbeck JP, Ronquist F (2001). MRBAYES: bayesian inference of phylogenetic trees. Bioinformatics.

[ref-43] James MT (1967). The blow flies of Dominica (Diptera: Calliphoridae). Proceedings of the Entomological Society of Washington.

[ref-44] James MT (1970). A catalogue of the Diptera of the Americas south of the United States: family Calliphoridae. Museu De Zoologica, Universidade De Sao Paulo.

[ref-45] Jordaens K, Sonet G, Braet Y, De Meyer M, Backeljau T, Goovaerts F, Bourguignon L, Desmyter S (2013a). DNA barcoding and the differentiation between North American and West European Phormia regina (Diptera, Calliphoridae, Chrysomyinae). Zookeys.

[ref-46] Jordaens K, Sonet G, Richet R, Dupont E, Braet Y, Desmyter S (2013b). Identification of forensically important *Sarcophaga* species (Diptera: Sarcophagidae) using the mitochondrial COI gene. International Journal of Legal Medicine.

[ref-47] Katoh K, Misawa K, Kuma K, Miyata T (2002). MAFFT: a novel method for rapid multiple sequence alignment based on fast Fourier transform. Nucleic Acids Research.

[ref-48] Kearse M, Moir R, Wilson A, Stones-Havas S, Cheung M, Sturrock S, Buxton S, Cooper A, Markowitz S, Duran C, Thierer T, Ashton B, Meintjes P, Drummond A (2012). Geneious basic: an integrated and extendable desktop software platform for the organization and analysis of sequence data. Bioinformatics.

[ref-49] Liu Q-L, Cai J-F, Chang Y-F, Gu Y, Guo Y-D, Wang X-H, Weng J-F, Zhong M, Wang X, Yang L, Wu K-L, Lan L-M, Wang J-F, Chen Y-Q (2011). Identification of forensically important blow fly species (Diptera: Calliphoridae) in China by mitochondrial cytochrome oxidase I gene differentiation. Insect Science.

[ref-50] Maddison DR, Maddison WP (2010a). http://mesquiteprojectorg/packages/chromaseq.

[ref-51] Maddison WP, Maddison DR (2010b). http://mesquiteprojectorg.

[ref-52] Masters BC, Fan V, Ross HA (2011). Species delimitation—a geneious plugin for the exploration of species boundaries. Molecular Ecology Resources.

[ref-53] Matuszewski S, Szafalowicz M, Jarmusz M (2013). Insects colonising carcasses in open and forest habitats of Central Europe: search for indicators of corpse relocation. Forensic Science International.

[ref-54] McDonagh L, Garcia R, Stevens JR (2009). Phylogenetic analysis of New World screwworm fly, *Cochliomyia hominivorax*, suggests genetic isolation of some Caribbean island populations following colonization from South America. Medical and Veterinary Entomology.

[ref-55] Meyer CP, Paulay G (2005). DNA barcoding: error rates based on comprehensive sampling. PLOS Biology.

[ref-56] Miller MA, Pfeiffer W, Schwartz T (2010). Creating the CIPRES Science Gateway for inference of large phylogenetic trees.

[ref-57] Nelson LA, Wallman JF, Dowton M (2007). Using COI barcodes to identify forensically and medically important blowflies. Medical and Veterinary Entomology.

[ref-58] Nelson LA, Wallman JF, Dowton M (2008). Identification of forensically important *Chrysomya* (Diptera : Calliphoridae) species using the second ribosomal internal transcribed spacer (ITS2). Forensic Science International.

[ref-59] Peris SV, Gonzalez-Mora D, Fernandez AM, Peris SJ (1998). A new species of *Calliphora* Robineau-Desvoidy, 1830 (Diptera, Calliphoridae) from Sierra Maestra, Cuba. Boletin De La Real Sociedad Espanola De Historia Natural Seccion Biologica.

[ref-60] Picard CJ, Wells JD (2009). Survey of the genetic diversity of phormia regina (Diptera: Calliphoridae) using amplified fragment length polymorphisms. Journal of Medical Entomology.

[ref-61] Posada D, Buckley TR (2004). Model selection and model averaging in phylogenetics: advantages of akaike information criterion and Bayesian approaches over likelihood ratio tests. Systematic Biology.

[ref-62] Posada D, Crandall KA (1998). MODELTEST: testing the model of DNA substitution. Bioinformatics.

[ref-63] Rambaut A, Drummond A (2009). Tracer v1.5. http://beast.bio.ed.ac.uk/Tracer.

[ref-64] Reibe S, Schmitz J, Madea B (2009). Molecular identification of forensically important blowfly species (Diptera: Calliphoridae) from Germany. Parasitology Research.

[ref-65] Rodrigo A, Bertels F, Heled J, Noder R, Shearman H, Tsai P (2008). The perils of plenty: what are we going to do with all these genes?. Philosophical Transactions of the Royal Society B-Biological Sciences.

[ref-66] Rosenberg NA (2007). Statistical tests for taxonomic distinctiveness from observations of monophyly. Evolution.

[ref-67] Shannon R (1926). Synopsis of the American Calliphoridae (Diptera). Proceedings of the Entomological Society of Washington.

[ref-68] Simon C, Frati F, Beckenbach A, Crespi B, Liu H, Flook P (1994). Evolution, weighting, and phylogenetic utility of mitochondrial gene-sequences and a compilation of conserved polymerase chain-reaction primers. Annals of the Entomological Society of America.

[ref-69] Smith KGV (1986). A manual of forensic entomology.

[ref-70] Solano JJ, Wolff M, Castrol LR (2013). Molecular identification of Calliphoridae (Diptera: Calliphoridae) of forensic importance in Colombia. Revista Colombiana De Entomologia.

[ref-71] Sonet G, Jordaens K, Braet Y, Desmyter S (2012). Why is the molecular identification of the forensically important blowfly species *Lucilia caesar* and *L. illustris* (family Calliphoridae) so problematic?. Forensic Science International.

[ref-72] Song ZK, Wang XZ, Liang GQ (2008). Species identification of some common necrophagous flies in Guangdong province, southern China based on the rDNA internal transcribed spacer 2 (ITS2). Forensic Science International.

[ref-73] Sukontason K, Sukontason KL, Piangjai S, Narongchai P, Samai W, Boonchu N, Sripakdee D, Ngern-klun R, Siriwattanarungsee S (2005). Morphology of second and third instars of *Chrysomya villeneuvi* Patton (Diptera : Calliphoridae), a fly species of forensic importance. Forensic Science International.

[ref-74] Szpila K, Hall MJR, Pape T, Grzywacz A (2013a). Morphology and identification of first instars of the European and Mediterranean blowflies of forensic importance. Part II. Luciliinae. Medical and Veterinary Entomology.

[ref-75] Szpila K, Hall MJR, Sukontason KL, Tantawi TI (2013b). Morphology and identification of first instars of the European and Mediterranean blowflies of forensic importance. Part I: Chrysomyinae. Medical and Veterinary Entomology.

[ref-76] Szpila K, Pape T, Hall MJR, Madra A (2014). Morphology and identification of first instars of European and Mediterranean blowflies of forensic importance. Part III: Calliphorinae. Medical and Veterinary Entomology.

[ref-77] Szpila K, Villet MH (2011). Morphology and identification of first instars of African blow flies (Diptera: Calliphoridae) commonly of forensic importance. Journal of Medical Entomology.

[ref-78] Tantawi TI, Whitworth T, Sinclair BJ (2017). Revision of the Nearctic *Calliphora* Robineau-Desvoidy (Diptera: Calliphoridae). Zootaxa.

[ref-79] Tarone AM, Singh B, Picard CJ, Tomberlin JK, Benbow EM (2015). Molecular biology in forensic entomology. Forensic entomology international dimensions and frontiers.

[ref-80] Tomberlin JK, Benbow EM (2015). Forensic entomology: international dimensions and frontiers.

[ref-81] Wallman JF, Donnellan SC (2001). The utility of mitochondrial DNA sequences for the identification of forensically important blowflies (Diptera : Calliphoridae) in southeastern Australia. Forensic Science International.

[ref-82] Wells JD, Byrd JH, Tantawi TI (1999). Key to third-instar *Chrysomyinae* (Diptera : Calliphoridae) from carrion in the continental United States. Journal of Medical Entomology.

[ref-83] Wells JD, Greenberg B (1992). Interaction between *Chrysomya rufifacies* and *Cochliomyia macellaria* (Diptera, Calliphoridae)—the possible consequences of an invasion. Bulletin of Entomological Research.

[ref-84] Wells JD, LaMotte LR, Byrd JH aCJ (2001). Estimating the postmortem interval. Forensic entomology: the utility of arthropods in legal investigations.

[ref-85] Wells JD, Wall R, Stevens JR (2007). Phylogenetic analysis of forensically important Lucilia flies based on cytochrome oxidase I sequence: a cautionary tale for forensic species determination. International Journal of Legal Medicine.

[ref-86] Wells JD, Williams DW (2007). Validation of a DNA-based method for identifying Chrysomyinae (Diptera : Calliphoridae) used in a death investigation. International Journal of Legal Medicine.

[ref-87] Whitworth T (2006). Keys to the genera and species of blow flies (Diptera : Calliphoridae) of America North of Mexico. Proceedings of the Entomological Society of Washington.

[ref-88] Whitworth T (2010). Keys to the genera and species of blow flies (Diptera: Calliphoridae) of the West Indies and description of a new species of *Lucilia* Robineau-Desvoidy. Zootaxa.

[ref-89] Whitworth T (2014). A revision of the Neotropical species of *Lucilia* Robineau-Desvoidy (Diptera: Calliphoridae). Zootaxa.

[ref-90] White TJ, Bruns T, Lee S, Taylor J, Dhg MA, Sninsky JJ, White TJ (1990). Amplification and direct sequencing of fungal ribosomal RNA genes for phylogenetics. PCR protocols: a guide to Methods and applications.

[ref-91] Whitworth T, Rognes K (2012). Identification of neotropical blow flies of the genus Calliphora Robineau-Desvoidy (Diptera: Calliphoridae) with the description of a new species. Zootaxa.

[ref-92] Williams KA, Lamb J, Villet MH (2016). Phylogenetic radiation of the greenbottle flies (Diptera, Calliphoridae, Luciliinae). Zookeys.

[ref-93] Williams K, Villet MH (2013). Ancient and modern hybridization between *Lucilia sericata* and *L. cuprina* (Diptera: Calliphoridae). European Journal of Entomology.

[ref-94] Woodley NE, Hilburn DJ (1994). The diptera of bermuda. Contributions of the American Entomological Institute.

[ref-95] Yusseff-Vanegas SZ (2007). Efectos de la temperatura sobre el desarrollo de *Chrysomya rufifacies* y *Cochliomyia macellaria* (Diptera: Calliphoridae), dos especies importantes para la entomología forense en Puerto Rico. Thesis M S.

[ref-96] Yusseff-Vanegas SZ (2014). Description of third instars of *Cochliomyia minima* (Diptera: Calliphoridae) from West Indies, and updated identification key. Journal of Medical Entomology.

[ref-97] Yusseff-Vanegas S, Agnarsson I (2016). Molecular phylogeny of the forensically important genus *Cochliomyia* (Diptera: Calliphoridae). Zookeys.

[ref-98] Zhang JJ, Kapli P, Pavlidis P, Stamatakis A (2013). A general species delimitation method with applications to phylogenetic placements. Bioinformatics.

[ref-99] Zwickl DJ (2006). Genetic algorithm approaches for the phylogenetic analysis of large biological sequence datasets under the maximum likelihood criterion. PhD dissertation.

