# Supplementary figures and images for: DNA-barcoding of forensically important blow flies (Diptera: Calliphoridae) in the Caribbean Region
Source: PeerJ. 2017 Jul 25;5:e3516. doi: 10.7717/peerj.3516 (PMC5531032; doi:10.7717/peerj.3516)

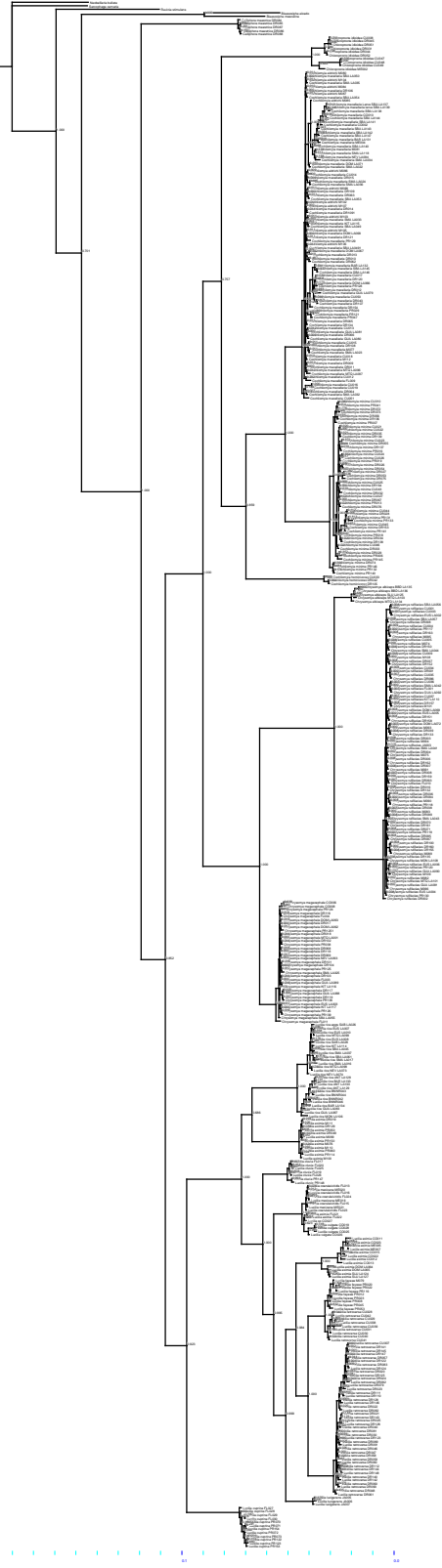

Supplement: Figure S1 — Numbers indicate posterior probability support values. Specimen voucher codes referred to in Table 1 are shown following species names. For specimens from Lesser Antilles (LA), the three capital letters before the voucher code refers to the name of the islands abbreviated a follows: SBA, St. Barthelemy; SAB, Saba; BAR, Barbuda; NEV, Nevis; KIT, St. Kitts; MTQ, Martinique; ANT, Antigua; GUA, Guadeloupe; MON, Montserrat; EUS, St. Eustatius; SMA, St. Martin, SLU, St. Lucia; BBD Barbados. [file peerj-05-3516-s001.pdf]

Calliphora maestrica DR084  
Calliphora maestrica DR086

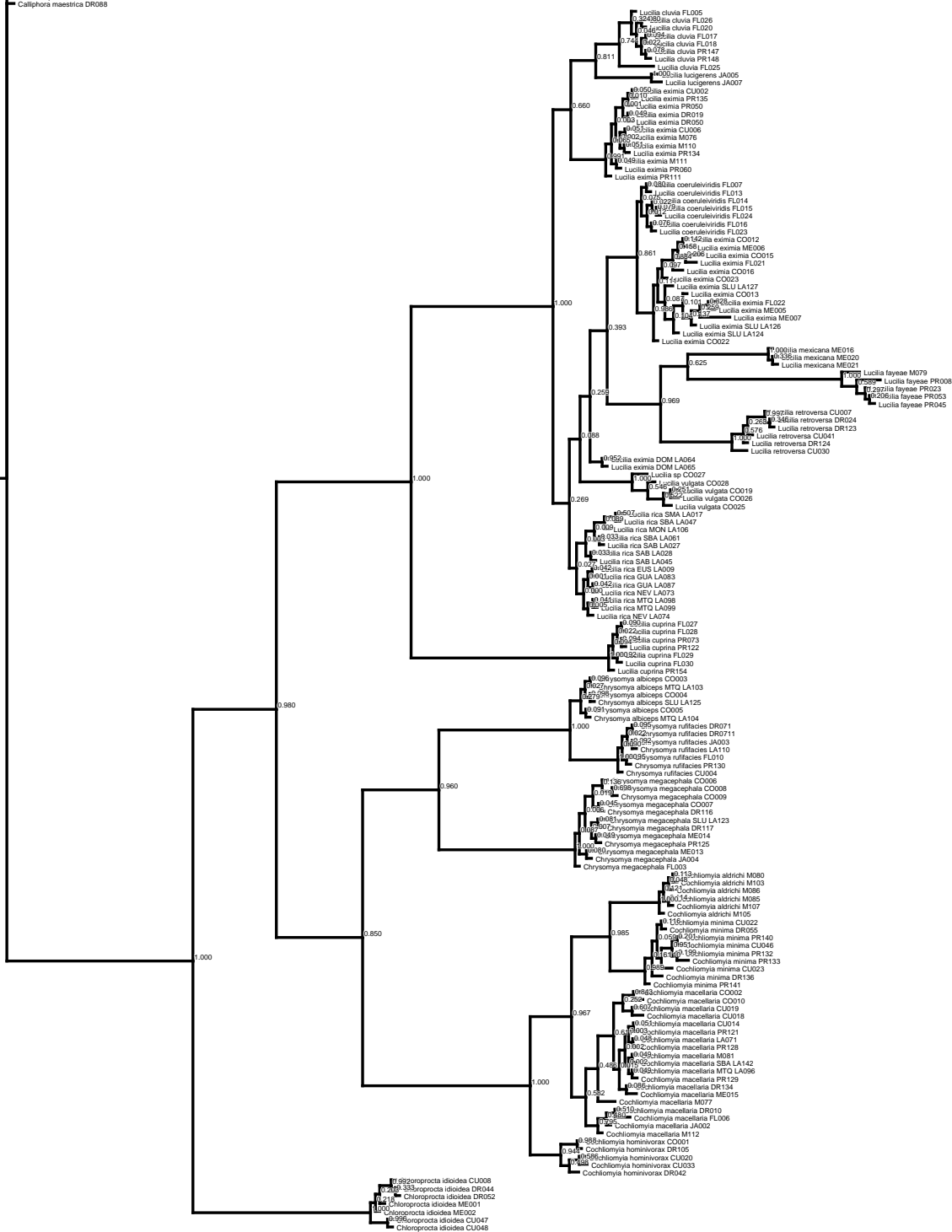

0.3

0.2

0.1

0.0

Supplement: Figure S2 — Numbers indicate posterior probability support values. Specimen voucher codes referred to in Table 1 are shown following species names. For specimens from Lesser Antilles (LA), the three capital letters before the voucher code refers to the name of the islands abbreviated a follows: SBA, St. Barthelemy; SAB, Saba; BAR, Barbuda; NEV, Nevis; KIT, St. Kitts; MTQ, Martinique; ANT, Antigua; GUA, Guadeloupe; MON, Montserrat; EUS, St. Eustatius; SMA, St. Martin, SLU, St. Lucia; BBD Barbados. [file peerj-05-3516-s002.pdf]
